# ECC-based three-factor authentication and key agreement scheme for wireless sensor networks

**DOI:** 10.1038/s41598-024-52134-z

**Published:** 2024-01-20

**Authors:** Wenfeng Huang

**Affiliations:** School of Information Engineering, Xiamen Ocean Vocational College, Xiamen, 361100 Fujian China

**Keywords:** Engineering, Mathematics and computing

## Abstract

In wireless sensor networks (WSNs), protocols with authentication and key agreement functions can enhance the security of the interaction between users and sensor nodes, guaranteeing the security of user access and sensor node information. Existing schemes have various security vulnerabilities and are susceptible to security attacks (e.g., masquerading user, password guessing, internal privilege, and MITT attacks), so they cannot meet the anonymity requirements or achieve forward security. To effectively improve the security performance of WSNs, an elliptic curve cryptography (ECC)-based three-factor authentication and key agreement scheme for WSNs is proposed. The scheme is based on the ECC protocol and combines biometrics, smart card and password authentication technology; uses a challenge/response mechanism to complete the authentication between users, gateways, and sensors; and negotiates a secure session key. The Burrows, Abadi and Needham logic for formal security analysis proves the correctness and security of the scheme, and the informal analysis of multiple known attacks proves that the scheme can resist various attacks and has high security characteristics. The feasibility of the scheme has been analysed and verified with the ProVerif tool. The efficiency analysis results show that the scheme is suitable for resource-constrained WSNs.

## Introduction

As wireless sensor networks (WSNs) are widely used in various application areas, securing their communication has become one of the focuses of researchers. The confidentiality of information communication is a major challenge, and protecting the privacy of data from unauthorized access by attackers is a major problem facing Internet of Things (IoT) WSNs^1^. Current schemes suffer from various security vulnerabilities in authentication and key agreement functions and are susceptible to security attacks such as masquerading users, password guessing, insider privileges, and MITM (Man-in-the-Middle), so they cannot satisfy anonymity requirements or achieve forward security. In IoT WSNs, establishing user authentication protocols with session keys is an approach that is widely used to solve the above problems. In this context, this study aims to address the security vulnerabilities in existing WSNs, especially in the interaction between users and sensor nodes, to ensure the security of user access and sensor node information.

The significance of this research lies in the following points: (1) Safeguarding communication security: WSNs are widely used in environmental monitoring, health care, intelligent transportation, etc., which include data communication that often involves personal privacy and important information. By improving the security of authentication and key agreement, this study helps to secure user access and sensor node information against potential attack risks. (2) Filling existing security holes: In this study, it is found that there are various vulnerabilities in the current security protocols in WSNs, which may be subject to attacks such as camouflage and password guessing. By combining elliptic curve cryptography and multifactor authentication techniques, this scheme is expected to fill these loopholes and improve the overall security of WSNs. (3) Promotion of the development of security in the field of WSNs: With the evolution of the IoT, the range of applications of WSNs is expanding. Research on communication schemes with high security is crucial for the healthy development of WSNs. This study aims to offer fresh insights and approaches for enhancing security in WSNs. (4) Positive impact on practical applications: Not only is the correctness and security of the scheme verified through formal BAN logic and the ProVerif tool, but its ability to fight against a wide range of attacks through informal analysis is also verified. This makes the scheme more likely to succeed in practical applications and provides strong technical support for real-world deployments. (5) Suitable for resource-constrained environments: The results of the efficiency analysis show that the scheme is suitable for resource-constrained WSNs. This is a substantial advantage for sensor nodes that have limited computational and storage resources and is expected to have a positive impact in the real world.

To effectively enhance the security performance of WSNs, this study proposes a three-factor authentication and key agreement scheme based on elliptic curve cryptography (ECC). The scheme is based on the ECC protocol, combines biometric, smart card and cryptographic authentication techniques, uses a challenge/response mechanism to complete the authentication between the user, the gateway and the sensor, and negotiates a secure session key. The correctness and security of the scheme are validated through formal security analysis using BAN logic. In addition, the scheme is verified as highly secure against various attacks through informal analysis of a variety of known attacks. To ensure the feasibility of the research, the paper also provides an exhaustive analysis and validation of the scheme using the ProVerif tool. The final efficiency analysis results show that the scheme is suitable for resource-constrained WSNs and provides a feasible and efficient solution for secure communication in WSNs. The purpose of this study is to promote the development of security in the field of WSNs and to provide a more reliable protection mechanism for wireless sensor networks in practical applications.

### Related works

In 2015, Lee et al.^2^ proposed a nontamper smart card authentication key protocol scheme based on anonymous passwords. In 2017, Wu et al.^3^ noted that the scheme of Lee et al.^2^ is not resistant to smart card loss, spoofed users, spoofed server attacks, and so forth. Wu et al. proposed an enhanced anonymous password authentication key agreement scheme. In 2016, Jiang et al.^4^ proposed a two-factor authentication scheme based on ellipse curve cryptography (ECC) for untraceable time vouchers in WSNs. In 2018, Li et al.^5^ found flaws in the work of Jiang et al.^4^, such as the lack of a password detection and change mechanism and a clock synchronization problem. Thus, Li et al. proposed a three-factor anonymous authentication scheme for WSNs in the IoT environment, using a fuzzy commitment scheme and error correction code to process user biometric information; however, the scheme proved to be unable to resist smart card loss attacks and achieve forward security. In 2022, Meriam et al.^6^ performed an informal security analysis of the protocol of Li et al.^5^, and the results showed that it cannot achieve anonymity and cannot resist session key leakage, internal, and other attacks. Thus, Meriam et al. proposed a three-factor mutual authentication and key agreement protocol for IoT WSNs based on lightweight ECC, using physically unclonable functions (PUFs) and ECC to improve security and effectively solve the security problem of Li et al.’s proposal^5^.

In 2017, Wu et al.^7^ proposed a user authentication scheme for WSNs based on the Internet of Things(IoT) and, in the same year, an efficient authentication and key agreement scheme for multigateway WSNs in the deployment of the IoT^8^. In 2019, Bayat et al.^9^ noted that the scheme of Wu et al.^7^ could not withstand certain security attacks. Thus, Bayat et al. proposed an analysis and improvement of the user authentication scheme of the IoT based on ECC. In 2019, Guo et al.^10^ found that the scheme of Wu et al.^8^ was inefficient and instead proposed a secure and efficient three-factor multigateway authentication protocol for WSNs; however, this scheme proved to be unable to resist offline password guessing and other attacks. In 2017, Jung et al.^11^ proposed an efficient and secure anonymous authentication scheme based on key agreement in WSNs. In the same year, Sravani et al.^12^ proposed an authentication key establishment scheme based on a secure signature for future IoT applications. However, the scheme was not resistant to man-in-the-middle attacks and was too complex and inefficient^13^.

In 2021, Azrour et al.^14^ proposed a new, enhanced IoT authentication protocol based on the literature^2,5^, and^9^, that could resist replay, internal, and other attacks. In 2021, Vinoth et al.^15^ proposed a multifactor authentication key protocol scheme for industrial IoT security; however, this scheme could not deal with certain types of attacks, such as sensor node capture and replay attacks. In 2021, Xue et al.^16^ proposed a lightweight three-factor authentication and key agreement scheme for multigateway WSNs in the IoT based on a ummary of the literature^10,14^, and ^15^ and proved the correctness and security of the proposed scheme through the BAN logic and BPR model. However, the scheme could not guarantee the security of the user's private key or negotiate a secure session key.

### Motivation, contributions and road-map

#### Motivation

The motivation of this paper is to improve the security of wireless sensor networks (WSNs), especially to enhance the authentication and key agreement features in the interaction between users and sensor nodes. Currently existing schemes suffer from various security vulnerabilities and are susceptible to security attacks such as masquerading users, password guessing, internal privileges, and man-in-the-middle attacks. These vulnerabilities make it difficult for existing schemes to meet anonymity requirements and achieve forward security. In this article, they propose an integrated authentication and key agreement scheme based on the ECC protocol is proposed, combining multiple authentication techniques to improve the security performance of WSNs, and demonstrate its feasibility and high level of security through formal and informal security analysis.

#### Their contribution

1. This paper proposes a three-factor authentication and key agreement scheme based on ECC for WSNs^17^. The new scheme is based on the ECC key agreement mechanism and introduces the challenge/response mechanism to establish authentication and key agreement mechanisms among users and gateways and sensors of WSNs. The security of the scheme is guaranteed by the security characteristics of biometrics, the elliptic curve discrete logarithm problem, and the one-way characteristics of the hash function.
2. After the authentication and key agreement between the user and the sensor is completed, a password update and smart card logout scheme is proposed to assist users in better managing smart cards and enhance the security of the scheme.
3. The proposed scheme is validated in several forms. The scheme's security is assessed through a formal analysis employing BAN logic. In addition, the nonformal security analysis proves the security performance of the scheme and its resistance to various attacks. Furthermore, simulations using the ProVerif tool validate the feasibility of the proposed scheme. Finally, the performance analysis shows that the scheme improves security without increasing energy consumption.

#### The road-map of the paper is as follows

In Section “Mathematical preliminaries”, they reviewed some of the basics of math and information security and defined the notations and descriptions and threat model used by the scheme. In Section “Safety analysis of existing schemes”, the advantages and some security vulnerabilities in the work of Xue et al.^16^ are discussed. Sections “The proposed scheme” and “Security analysis” present the proposed scheme and the corresponding security analysis, respectively. In Section “Efficiency analysis”, the performance of the proposed scheme is evaluated, and finally, the whole paper is concluded in Section “Conclusions”.

## Mathematical preliminaries

### Cryptanalysis

Cryptanalysis, a subset of cryptography, is the process of deciphering or breaking cryptographic systems. It utilizes techniques such as mathematics, computer science, and engineering to unveil encrypted data. The primary objective of cryptanalysis is to achieve unauthorized access to encrypted information by scrutinizing weaknesses in encryption algorithms, key management, and security mechanisms. This involves activities such as password guessing, analysing the mathematical aspects of encryption algorithms, identifying vulnerabilities in encryption keys, and exploiting errors in implementation. The efficacy of cryptanalysis hinges on the intricacy and robustness of the cryptosystem. This field plays a pivotal role in information security, contributing to the evaluation and enhancement of cryptographic system strength.

### ECC and ECDH^18^

Elliptic Curve Cryptography (ECC) is a public key encryption algorithm that is widely used in the field of cryptography. The security of ECC is based on the discrete logarithmic problem on elliptic curves, which is considered to be difficult to solve; thus, encryption algorithms based on this mathematical puzzle provide a high level of security. Compared to traditional RSA algorithms based on the integer factorization problem, ECC can use shorter key lengths while providing the same level of security, thus reducing the computational and storage requirements. Overall, elliptic curve cryptography is an important part of the modern field of cryptography and provides a powerful tool for secure communication.

The elliptic Curve Diffie-Hellman key exchange (ECDH) is mainly used to establish secure shared encryption data in an insecure channel, generally exchanging private keys, which are generally used as "symmetric encryption" keys by both parties for subsequent data transmission. ECDH is based on the premise that given a point P on an elliptic curve and an integer k, it is easy to solve for Q = KP, but it is difficult to solve for K via Q, P.

### BAN logic

BAN logic is a formal method for analysing and verifying cryptographic schemes, proposed by Burrows, Abadi, and Needham (BAN) in 1989^19^. The basic idea of BAN logic is to convert messages in a cryptographic scheme into a logical language representation and then use inference rules to derive the beliefs and goals of the participants in the scheme. BAN logic can be used to find vulnerabilities in a scheme to improve its security and efficiency.

Table 1 shows the notations used by BAN logic^20^ and descriptions of these notations. The BAN logic rules used include: message meaning rule R1: $\frac{{P| \equiv P\mathop \leftrightarrow \limits^{SK} Q,P \triangleleft \left\{ H \right\}_{SK} }}{{P\left| { \equiv Q} \right|{\sim }H}}$, random number verification rule R2: $\frac{{P\left| { \equiv \# \left( H \right),P} \right| \equiv Q|{\sim }H}}{{P\left| { \equiv Q} \right| \equiv H}}$, arbitration rule R3: $\frac{{P\left| { \equiv Q} \right| \equiv H,P\left| { \equiv Q} \right| \Rightarrow H}}{P| \equiv H}$, freshness rule R4: $\frac{P| \equiv \# \left( H \right)}{{P| \equiv \# \left( {H,G} \right)}}$, belief rule R5: $\frac{{P| \equiv \left( {H,G} \right)}}{P| \equiv G}$, and session secret key rule R6: $\frac{{P\left| { \equiv \# \left( H \right),P} \right| \equiv Q| \equiv H}}{{P| \equiv P\mathop \leftrightarrow \limits^{SK} Q}}$.

**Table 1:** Notations used by BAN logic and descriptions of these notations.

Notations	Descriptions	Notations	Descriptions
$P| \equiv H$	P believes H is true	$P\mathop \leftrightarrow \limits^{SK} Q$	Both P and Q can use the shared key SK to communicate with each other
$P \triangleleft H$	P sees H and is capable of reading and repeating it	$P|{\sim }H$	P once said H; at some time, P has sent the message containing H
$\# \left( H \right)$	H is fresh which means it was never sent before the current execution of the protocol	$P| \Rightarrow H$	P has control or jurisdiction over H
$\left\{ H \right\}_{K}$	The ciphertext obtained by encrypting plaintext H with key K		

### Random oracle model

In 1993, Bellare and Rogaway formally proposed the Random Oracle Model (ROM) methodology, with which the past purely theoretical research of provable security methodology quickly made significant progress in the field of practical applications. A large number of fast and effective security programs have been proposed, and at the same time, they also produced the "concrete security or exact security", which means that they no longer only satisfy the asymptotic degree of security but can exactly obtain a more accurate security measure. Practical-oriented provable security theory has been widely accepted by academia and industry.

Inside cryptography, a random oracle is a prediction machine (simply put, like a black box for the theory) that returns a truly uniformly random output for any input, and for the same input, this prediction machine outputs the same output in the same way every time (i.e., if the query is repeated, it responds in the same way every time the query is submitted). In other words, a randomized prediction machine is a function that randomly maps all possible inputs to outputs.

The stochastic prediction machine model is usually an idealized stand-in for the real hash function and has its origins in the idea of viewing hash functions as pseudorandom. The stochastic prediction machine model has the following properties:

1. Consistency: Inputs that are the same should produce matching outputs.
2. Computability: the output can be calculated within a polynomial time frame.
3. Uniform Distributability: The prediction machine's output is evenly spread across the value space without any overlaps.
4. In the stochastic prediction machine model, it is assumed that the adversary will not exploit the weakness of the hash function to attack the cryptographic scheme.

## Notations and descriptions

Table 2 shows the notations used in this paper and descriptions of these notations.

**Table 2:** Notations used in this paper and descriptions of these notations.

Notations	Descriptions	Notations	Descriptions
*U*_*i*_ and *U*_*a*_	User *U*_*i*_ and *U*_*a*_	*E*(*F*_*p*_)	Elliptic curve finite field *E(F*_*p*_*)*
*GWN*	Gateway node *GWN*	*P*	Base points on elliptic curves *P*
*S*_*j*_	Sensor node *S*_*j*_	*r*_*i*_ and *r*_*u*_	The private key *r*_*i*_ and *r*_*u*_ of the user *U*_*i*_
*ID*_*i*_	The identity *ID*_*i*_ of the user *U*_*i*_	*r*_*g*_	The private key *r*_*g*_ of the gateway node *GWN*
*ID*_*hg*_	The identity *ID*_*hg*_ of the gateway node *GWN*	*r*_*s*_	The private key *r*_*s*_ of the sensor node *S*_*j*_
*SID*_*j*_	The identity *SID*_*j*_ of the sensor node *S*_*j*_	*R*_*i*_ and *R*_*u*_	The public key *R*_*i*_ and *R*_*u*_ of the user *U*_*i*_
*PW*_*i*_	The password *PW*_*i*_ of the user *U*_*i*_	*R*_*g*_	The public key *R*_*g*_ of the gateway node *GWN*
*BIO*_*i*_	The biological factor *BIO*_*i*_ of the user *U*_*i*_	*R*_*s*_	The public key *R*_*s*_ of the sensor node *S*_*j*_
*SC*_*i*_	The smart card *SC*_*i*_ of the user *U*_*i*_	·	Elliptic curve point multiplication operation
*SK*_*u*_	The negotiated session key *SK*_*u*_ of the user *U*_*i*_	*Gen*	The generation process of fuzzy extraction
*SK*_*s*_	The negotiated session key *SK*_*s*_ of the sensor node *S*_*j*_	*Rep*	Recovery process of fuzzy extraction
*K*_*G*_	Gateway node secret value *K*_*G*_	*α*_*i*_	The random secret information generated by fuzzy extraction *α*_*i*_ of the user *U*_*i*_
*List*	Number of user authentication	*β*_*i*_	The auxiliary bit string generated by fuzzy extraction *β*_*i*_ of the user *U*_*i*_
‖	concatenation operator	*h*(*·*)	hash function
*T*	Timestamp	⊕	XOR operator
*ΔT*	Maximum permitted transmission delay	*mod*	Modular exponentiation

### Threat model^18^

In this article, the following threat models are used:

1. Communication conducted over a public channel is susceptible to eavesdropping, providing attackers with an advantage.
2. Threats to any system can come from external entities or even legitimate users who may act as attackers.
3. Attackers have the capability to manipulate, erase, redirect, and replay intercepted messages, compromising the integrity of the communication.
4. The attacker is assumed to possess knowledge of the protocol used in the authentication system.

## Safety analysis of existing schemes^16^

Scheme^16^ proposed an authentication and key agreement scheme for multigateway environments. In the scheme, biometrics, a crucial element, is extracted and authenticated using a fuzzy extractor. The program consists of the following six processes:

1. System initialization. The *SA* assigns identity *ID*_*hg*_, *ID*_*fg*_ and private keys *x*_*hg*_, *x*_*fg*_ to *HGWN* and *FGWN* and establishes a shared key *K*_*hf*_. The *HGWN* and *FGWN* independently choose three random numbers, denoted as *R*_*h*_, *R*_*f*_ and *R*_*fh*_, respectively.
2. Registration. This stage comprises sensor registration and user registration. Both sensor nodes and users are needed to register their fundamental details with the nearest *HGWN* gateway. After the registration, *U*_*i*_ saves *B*_*1*_ = *h*(*α*_*i*_‖*ID*_*i*_‖*PW*_*i*_) ⊕ *r*_*i*_, *B*_*2*_ = *h*(*HPW*_*i*_‖*α*_*i*_‖*ID*_*i*_‖*r*_*i*_*)mod n*_0_ to *SC*, *HGWN* saves *SID*_*j*_, and *S*_*j*_ saves *x*_*j*_.
3. Login. *U*_*i*_ inputs *ID*_*i*_, *PW*_*i*_, and *BIO*_*i*_, *SC* verifies the identity of *U*_*i*_ by calculating *B*_2_ = *h*(*HPW*_*i*_*‖α*_*i*_‖*ID*_*i*_‖*r*_*i*_)*mod n*_0_, if the verification passes, *U*_*i*_ sends *M*_1_ = {*TID*_*i*_, *ID*_*hg*_, *SID*_*j*_, *D*_0_, *D*_1_, *D*_2_, *D*_3_, *T*_1_} over the public channel to *HGWN*.
4. Authentication and key agreement.After receiving the communication request between *U*_*i*_ and *SID*_*j*_, *HGWN* initially verifies if the designated sensor *S*_*j*_ is within its communication range. If *HGWN* can retrieve *SID*_*j*_ from its local database, it can proceed following Case 1, and the three parties, *U*_*i*_, *HGWN*, and *SID*_*j*_, perform authentication and key agreement; otherwise, it operates according to Case 2, and the four parties, *U*_*i*_, *HGWN*, *FGWN*, and *SID*_*j*_, perform authentication and key agreement.
5. Password update. User enters his or her *ID*_*i*_, *PW*_*i*_, and *BIO*_*i*_, and *SC* verifies. If the verification passes, the user enters new password *PW*_*i*_', *SC* computes new *B*_1_^′^, *B*_2_^′^, and *e*_*i*_^′^ and saves.
6. Smart card logout. The user enters his or her *ID*_*i*_, *PW*_*i*_, and *BIO*_*i*_ and *SC* verifies it. If the verification passes, *U*_*i*_ sends *M*_0_ = {*TID*_*i*_, *β*_*i*_, *R*_0_, *T*_1_} over the public channel to *HGWN*. *HGWN* verifies that *K*_*i*_' is equal to *K*_*i*_ by computation. if the verification passes it deletes *U*_*i*_'s information *{ID*_*i*_*, K*_*i*_*, honey_list}*.

The existing scheme^16^ has some advantages in resisting password guessing, replay, and other attacks to achieve two-way authentication and key agreement; however, there are also security vulnerabilities, such as the inability to guarantee anonymity and the potential to suffer from MITT attacks. In this section, the advantages of the scheme and the existence of security vulnerabilities are presented^21^.

### Advantages of the scheme^16^

The advantages of the schemes^16^ include the following:

1. The use of biometric-based fuzzy extraction technology effectively enhances the security of user login via the three-factor authentication mechanism.
2. Security of the authentication process is ensured through use of the challenge/response mechanism^22^.
3. The user’s secret *x*_*i*_ and the sensor’s secret *x*_*j*_ are calculated using the hash function, and they are not transmitted in the public channel, which can prevent the secret from being cracked and ensure its forward security.
4. The honey list technique, which can prevent password guessing attacks by setting the number of logins and avoid smart card loss attacks and offline guessing attacks, is adopted.
5. Replay attacks are avoided by setting the timestamp *T*.
6. Two-way authentication and key agreement are achieved as the negotiated session key *SK* contains a random number of users, gateways, and sensors to improve the security of the negotiated key^23^.

### Security vulnerabilities of the scheme^16^

The scheme’^16^ security vulnerabilities include the following:

1. Unable to meet the anonymity requirement: During the registration process, *U*_*i*_ sends *ID*_*i*_ to *HGWN*, *Sj* sends *SID*_*j*_ to *HGWN*, and *HGWN* sends *ID*_*hg*_ to *U*_*i*_. Attackers intercept *ID*_*i*_, *ID*_*hg*_, and *SID*_*j*_ in the public channel to easily obtain the identity *ID*_*s*_ of the user, gateway, and node. Therefore, the scheme cannot guarantee anonymity.
2. Unable to secure user parameters^24^: During the registration process, *U*_*i*_ sends {*ID*_*i*_, *HPW*_*i*_, *β*_*i*_} to the *HGWN*. The attacker intercepts *ID*_*i*_ in the public channel. During the login process, *U*_*i*_ sends *M*_1_ = {*TID*_*i*_, *ID*_*hg*_, *SID*_*j*_, *D*_0_, *D*_1_, *D*_2_, *D*_3_, *T*_1_} to the *HGWN*. The attacker intercepts *D*_*2*_ in the public channel and calculates:
  1 $$h(r_{u} ||x_{i} ) = ID_{i} \oplus D_{2}$$
  The attacker intercepts *D*_*0*_ and calculates:
  2 $$\beta_{i} = D_{0} \oplus h(x_{i} ||r_{u} )$$
  3 $$K_{i} = h(ID_{i} ||\beta_{i} )$$
  4 $$e_{i} = HPW_{i} \oplus K_{i} \oplus x_{i}$$
  The attacker obtains all the parameters of the user login.
3. Unable to secure user secrets *x*_*i*_ and sensor secrets *x*_*j*_: During the registration process, *U*_*i*_ sends {*ID*_*i*_, *HPW*_*i*_, *β*_*i*_} to *HGWN* and *HGWN* sends {*TID*_*i*_*, β*_*i*_*, e*_*i*_*, ID*_*hg*_} to *U*_*i*_. The attacker intercepts *HPW*_*i*_, *ID*_*i*_, *β*_*i*_, and *e*_*i*_ in the public channel and calculates:
  5 $$K_{i} = h(ID_{i} ||\beta_{i} )$$
  6 $$x_{i} = HPW_{i} \oplus K_{i} \oplus e_{i}$$
  The user secret *x*_*i*_ is cracked. Attackers directly obtain sensor secret *x*_*j*_ in the public channel.
4. Unable to secure user private key *r*_*u*_: During the login process, *U*_*i*_ sends *M*_1_{*TID*_*i*_, *ID*_*hg*_, *SID*_*j*_, *D*_0_, *D*_1_, *D*_2_, *D*_3_, *T*_1_} to *HGWN*, and the attacker intercepts *D*_1_ in the public channel and can crack *x*_*i*_ by point (3) above and calculates:
  7 $$r_{u} = D_{1} \oplus x_{i}$$
  The user private key *r*_*u*_ is cracked.
5. Unable to secure gateway private key *r*_*hg*_ and sensor private key *r*_*s*_: During the registration process, *HGWN* sends {*x*_*j*_} to *S*_*j*_. The attacker intercepts *x*_*j*_ in the public channel. During the authentication process, the *HGWN* sends *M*_2_ = {*D*_0_, *D*_4_, *D*_5_, *D*_6_, *T*_2_} to *S*_*j*_ and *S*_*j*_ sends *M*_3_ = {*D*_7_, *D*_8_, *T*_3_} to the *HGWN*. The attacker intercepts *D*_4_, *D*_7_, *T*_2_, *T*_4_ in the public channel and can crack^25^:
  8 $$r_{hg} = D_{4} \oplus h(x_{j} ||T_{2} )$$
  The attacker crack:
  9 $$r_{s} = D_{7} \oplus h(x_{j} ||r_{hg} ||T_{4} )$$
6. Unable to achieve secure two-way authentication: According to Points (2), (3), and (4) above, the attacker cracks *x*_*i*_, *r*_*u*_, *K*_*i*_, During the registration process, *U*_*i*_ sends {*ID*_*i*_, *HPW*_*i*_, *β*_*i*_} to the *HGWN*, and during the login process, *U*_*i*_ sends *M*_*1*_ = {*TID*_*i*_, *ID*_*hg*_, *SID*_*j*_, *D*_0_, *D*_1_, *D*_2_, *D*_3_, *T*_1_} to the *HGWN*. The attacker intercepts *TID*_*i*_, *ID*_*i*_, *SID*_*j*_, *T*_*1*_ in the public channel, and by calculating *D*_3_ = *h*(*TID*_*i*_‖*ID*_*i*_‖*SID*_*j*_‖*r*_*u*_‖*x*_*i*_‖*K*_*i*_‖*T*_1_) can crack *D*_3_, so the gateway authentication user algorithm is cracked. During registration, *HGWN* sends {*x*_*j*_} to *S*_*j*_, during login, *U*_*i*_ sends *M*_*1*_ = {*TID*_*i*_, *ID*_*hg*_, *SID*_*j*_, *D*_0_, *D*_1_, *D*_2_, *D*_3_, *T*_1_} to *HGWN*, and during authentication, *HGWN* sends *M*_2_ = {*D*_0_, *D*_4_, *D*_5_, *D*_6_, *T*_2_} to *S*_*j*_. According to Points (4) and (5) above, the attacker cracks *r*_*u*_, *r*_*hg*_ and intercepts *SID*_*j*_, *ID*_*hg*_, *x*_*j*_, *T*_2_ in the public channel; *D*_*6*_ can be cracked by calculating:
  10 $$D_{6} = h(SID_{j} ||ID_{hg} ||r_{u} ||r_{hg} ||x_{j} ||T_{2} )$$
  The sensor authentication gateway algorithm is cracked.
7. Unable to negotiate a secure session key: The negotiated key is *SK*_*s*_ = *h*(*r*_*u*_‖*r*_*hg*_‖*r*_*s*_‖*ID*_*hg*_). During the login process, *U*_*i*_ sends *M*_1_ = {*TID*_*i*_, *ID*_*hg*_, *SID*_*j*_, *D*_0_, *D*_1_, *D*_2_, *D*_3_, *T*_1_} to *HGWN*. According to Points (4) and (5) above, the attacker breaks *r*_*u*_, *r*_*hg*_, *r*_*s*_ and intercepts *ID*_*hg*_ in the public channel, which can crack:
  11 $$SK_{s} = h(r_{u} ||r_{hg} ||r_{s} ||ID_{hg} )$$
  The scheme cannot negotiate a secure session key, and it has forward security problems.
8. Unable to resist MITT attacks: The attacker records all *M*_1_ = {*TID*_*i*_, *ID*_*hg*_, *SID*_*j*_, *D*_0_, *D*_1_, *D*_2_, *D*_3_, *T*_1_} sent to the *GWN*, all *M*_2_ = {D_4_, *D*_5_, *D*_6_, *T*_2_} sent to *S*_*j*_, and all *x*_*j*_ sent to *S*_*j*_ by the gateway, and then calculates:
  12 $$r_{hg}^{*} = D_{4} \oplus h(x_{j}^{*} ||T_{2} )$$
  13 $$r_{u}^{*} = D_{5} \oplus h(r_{hg}^{*} ||x_{j}^{*} ||T_{2} )$$

For each group *M*_1_, the attacker calculates:

14 $$x_{i}^{*} = r_{u}^{*} \oplus D_{1}$$

15 $$\beta_{i}^{*} = D_{0} \oplus h(x_{i}^{*} ||r_{u}^{*} )$$

16 $$ID_{i}^{*} = D_{2} \oplus h(r_{u}^{*} ||x_{i}^{*} )$$

17 $$K_{i}^{*} = h(ID_{i}^{*} ||\beta_{i}^{*} )$$

Whether *D*_3_^*^ = *h*(*TID*_*i*_‖*ID*_i_^*^‖*SID*_*j*_‖*r*_u_^*^‖*x*_i_^*^‖*K*_i_^*^‖*T*_1_) is equal to *D*_*3*_ is verified. If equal, the attacker can determine user *U*_*i*_ with its corresponding *S*_*j*_ and obtain the values of the parameters *r*_*u*_, *x*_*i*_, and so on. The attacker starts a new session with user *U*_*i*_, selects *r*_*hg*_, *r*_*s*_, and *TID*_*i*_^′^, and calculates:

18 $$SK_{hg} = h(r_{u} ||r_{hg} ||r_{s} ||ID_{hg} )$$

19 $$D_{9} = r_{s} \oplus h(x_{i} ||r_{u} )$$

20 $$D_{10} = \, r_{hg} \oplus h(r_{u} ||x_{i} )$$

21 $$x_{i}^{{\prime }} = h(TID_{i}^{{\prime }} ||x_{hg} ) \oplus R_{h}$$

22 $$D_{11} = TID_{i}^{{\prime }} \oplus h(x_{i} ||ID_{i} ||r_{u} )$$

23 $$D_{12} = x_{i}^{{\prime }} \oplus h(TID_{i}^{{\prime }} ||x_{i} )$$

24 $$D_{13} = \, h(SK_{hg} ||x_{i}^{{\prime }} ||TID_{i}^{{\prime }} ||K_{i} ||T_{4} )$$

The attacker sends *M*_4_ = {*D*_9_, *D*_10_, *D*_11_, *D*_12_, *D*_13_, *T*_4_} to *U*_*i*_. *U*_*i*_ calculates:

25 $$r_{s}^{*} = D_{9} \oplus h(x_{i} ||r_{u} )$$

26 $$r_{hg}^{*} = \, D_{10} \oplus h(r_{u} ||x_{i} )$$

27 $$SK_{u}^{*} = h(r_{u} ||r_{hg}^{*} ||r_{s}^{*} ||ID_{hg} )$$

28 $$TID_{i}^{{{\prime * }}} = D_{11} \oplus h(x_{i} ||ID_{i} ||r_{u} )$$

29 $$x_{i}^{{{\prime }*}} = D_{12} \oplus h(TID_{i}^{{{\prime }*}} ||x_{i} )$$

*U*_*i*_ verifies whether *D*_13_^*^ = *h*(*SK*_hg_^*^‖*x*^′*^‖*TID*_*i*_^′*^‖*K*_*i*_‖*T*_4_) is equal to *D*_13_. If equal, according to the rule, the user accepts this *SK* as the agreement key and the attacker successfully implements the MITT attack.

## The proposed scheme

In this section, an ECC-based three-factor authentication and key agreement scheme for WSNs is proposed, the improvement measures of the scheme are introduced, and then a specific implementation scheme, including system initialization, node registration, user registration, two-way authentication and key agreement, password update, and smart card logout, is proposed^17^. The proposed scheme operates under the following security assumptions:

1. The gateway is securely impenetrable and has unlimited computation, storage, and communication capabilities.
2. The WSN network is a bidirectional channel, and nodes can communicate normally.
3. The WSN network employs asymmetric encryption, meaning it utilizes both public and private keys.
4. Upon successful completion of the key agreement in the WSN network, the user and the sensor node can establish communication using the session key.

### Scheme improvement measures

1. The authentication scheme is designed using an ECC key agreement protocol to ensure the forward security of the scheme.
2. The user *ID* is replaced by the user identifier *TID* after the hashing operation, all *ID*s are forbidden to be sent explicitly, and no direct XOR calculation can be performed to ensure the anonymity of the scheme.
3. Random numbers *r*_*u*_ and *r*_*s*_ are forbidden to be sent in clear text, and no direct XOR calculation can be performed to ensure secure two-way authentication and key agreement and resist MITT attacks^26^.
4. More complex parameters are selected to improve the security of the session key.
5. The relevant parameters in the *SC* card are updated after two-way authentication and key agreement to ensure that the scheme is resistant to internal attacks^27^.

### Specific implementation plan

**Figure 1:**
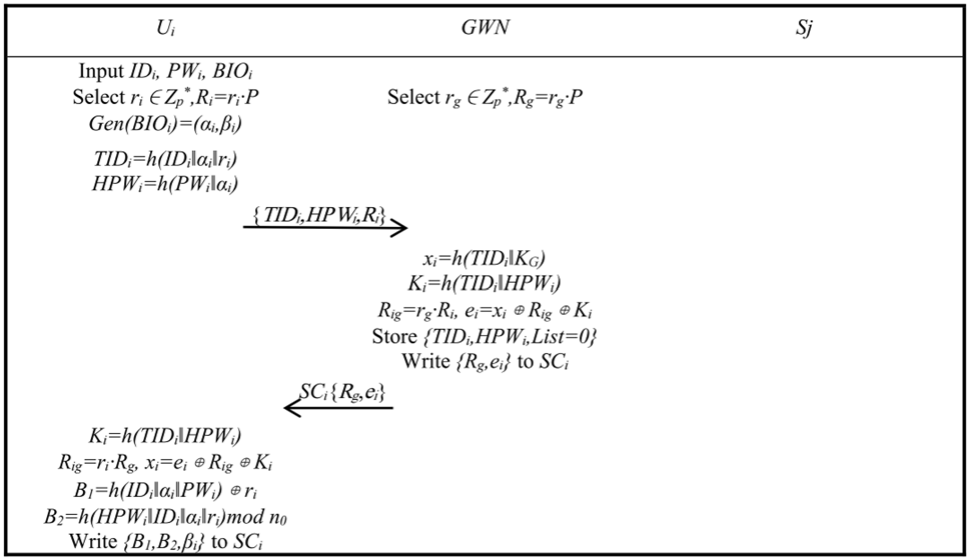
Registration phase.

**Figure 2:**
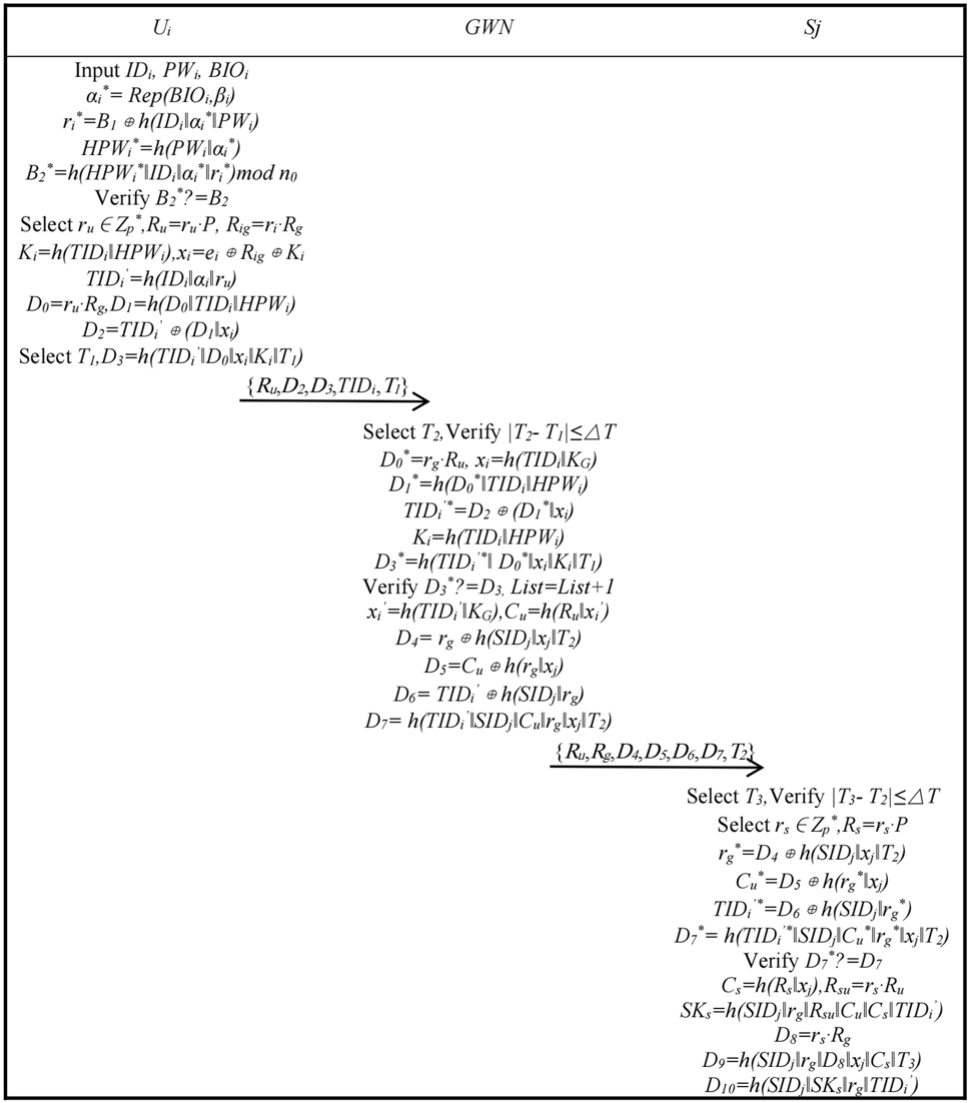
The authentication and key agreement phase 1.

**Figure 3:**
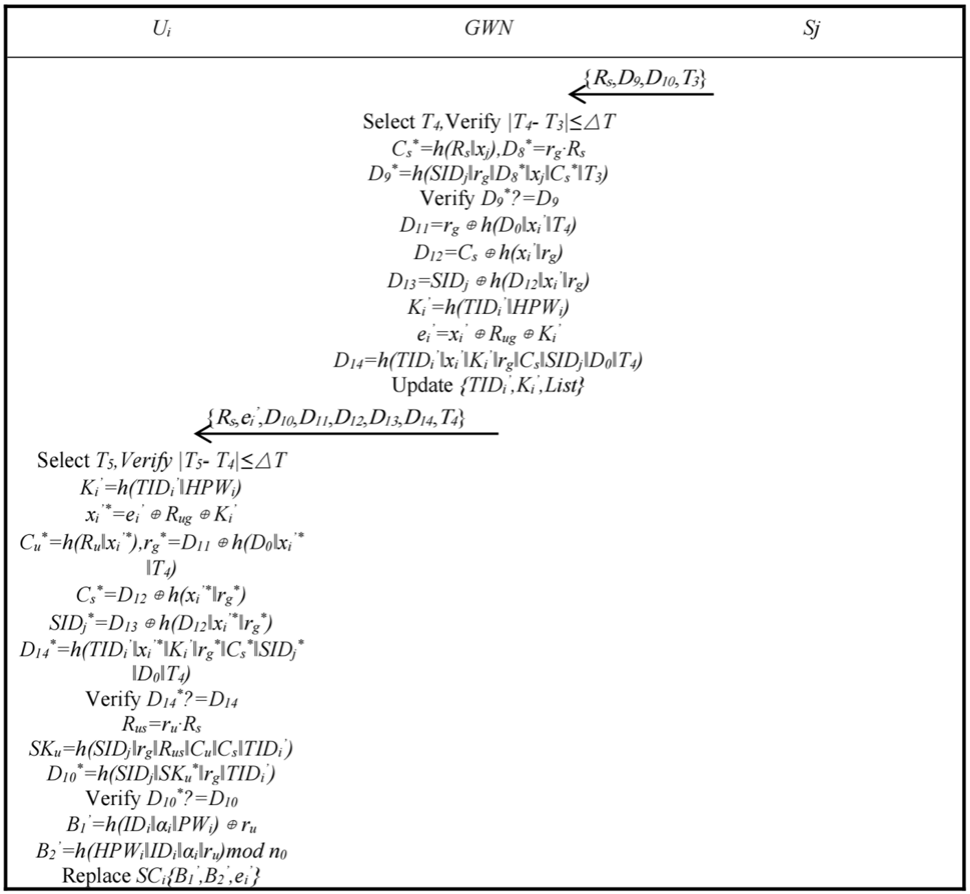
The authentication and key agreement phase 2.

**Figure 4:**
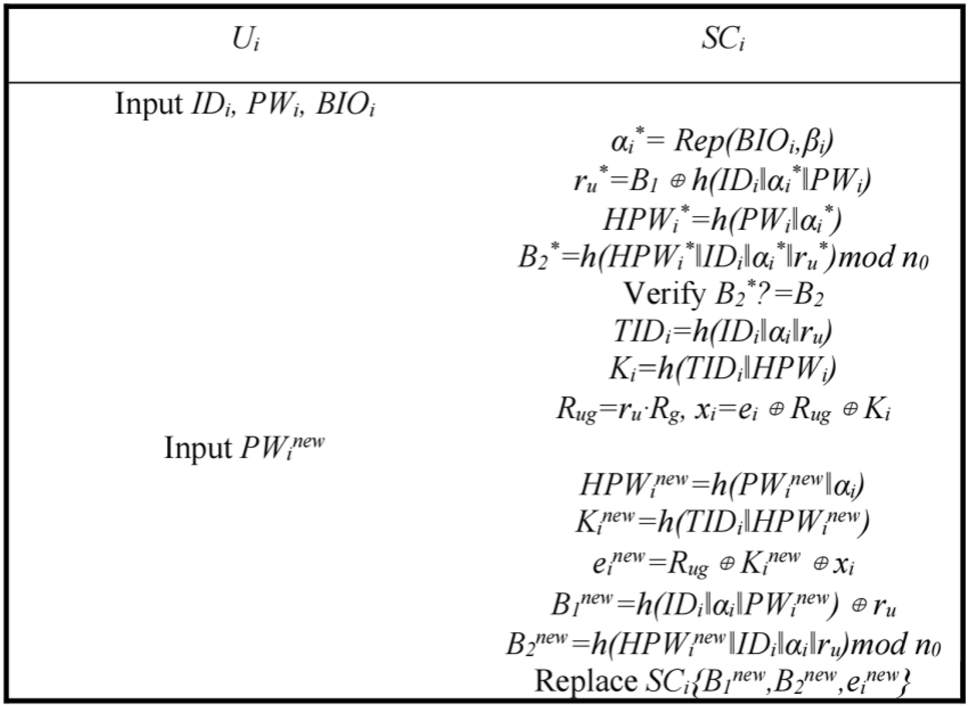
Password update.

**Figure 5:**
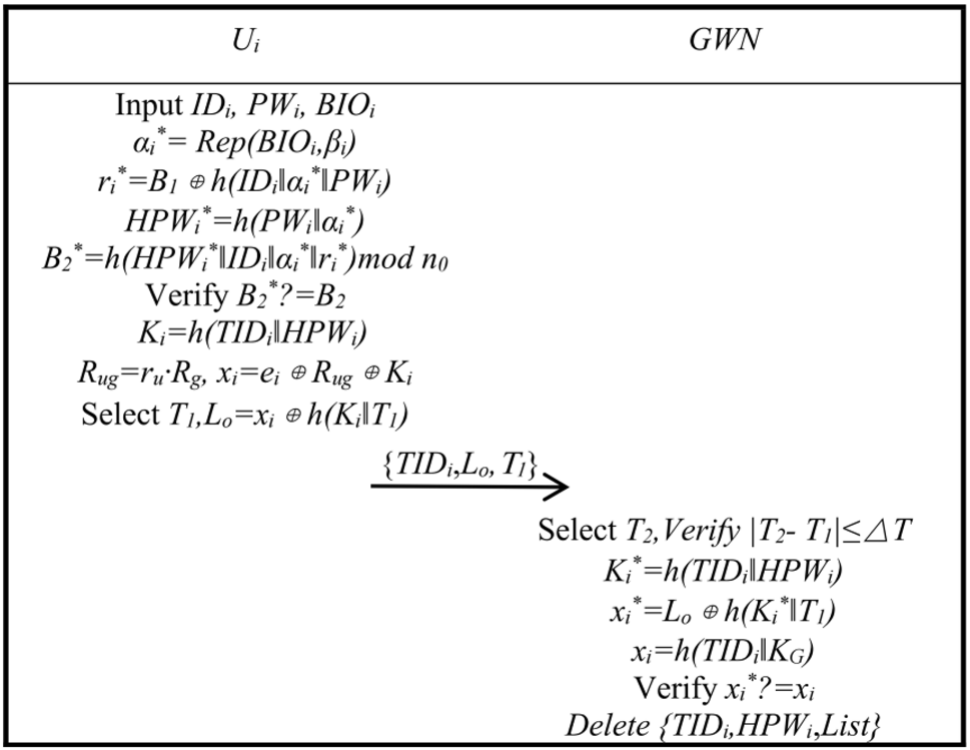
Smart card logout.

1. System Initialization
  At the very beginning, the system needs to be initialized. *GWN* selects *E*(*F*_*p*_), *P*, *h*(.) and the secret value *K*_*G*_, publicly release *E*(*F*_*p*_), *P*, *h*(.), save *K*_*G*_.
2. Node Registration
  After the system is initialized, the node can start registering. Node *S*_*j*_ applies for registration to the *GWN*, which selects the unique *SID*_*j*_ of the node, calculates *x*_*j*_ = *h*(*SID*_*j*_‖*K*_*G*_), and writes {*SID*_*j*_, *x*_*j*_} to node *S*_*j*_.
3. User Registration
  After the system is initialized, the user can start registering. The user registration process is shown in Fig. 1.
  Step R1: User *U*_*i*_ inputs *ID*_*i*_, *PW*_*i*_, *BIO*_*i*_, chooses random number *r*_*i*_ ∈ *Z*_p_^*^, calculates *R*_*i*_ = *r*_*i*_·*P*, *Gen*(*BIO*_*i*_) = (*α*_*i*_, *β*_*i*_), *TID*_*i*_ = *h*(*ID*_*i*_‖*α*_*i*_‖*r*_*i*_), *HPW*_*i*_ = *h*(*PW*_*i*_‖*α*_*i*_), and *U*_*i*_ sends {*TID*_*i*_, *HPW*_*i*_, *R*_*i*_} to *GWN*.
  Step R2: The gateway *GWN* chooses a random number *r*_*g*_ ∈ *Z*_p_^*^ and calculates *R*_*g*_ = *r*_*g*_·*P*. After the *GWN* receives the *U*_*i*_ message, it calculates *x*_*i*_ = *h*(*TID*_*i*_‖*K*_*G*_), *K*_*i*_ = *h*(*TID*_*i*_‖*HPW*_*i*_), *R*_*ig*_ = *r*_*g*_·*R*_*i*_, *e*_*i*_ = *x*_*i*_ ⊕ *R*_*ig*_ ⊕ *K*_*i*_, sets the number of logins *List* = 0, saves {*TID*_*i*_, *HPW*_*i*_, *List* = *0*}. Write {*R*_*g*_, *e*_*i*_} to smart card *SC*_*i*_ and issue to *U*_*i*_.
  Step R3: User *U*_*i*_ receives the smart card *SC*_*i*_, calculates *K*_*i*_ = *h*(*TID*_*i*_‖*HPW*_*i*_), *R*_*ig*_ = *r*_*i*_·*R*_*g*_, *x*_*i*_ = *e*_*i*_ ⊕ *R*_*ig*_ ⊕ *K*_*i*_, *B*_1_ = *h*(*ID*_*i*_‖*α*_*i*_‖*PW*_*i*_) ⊕ *r*_*i*_, *B*_2_ = *h*(*HPW*_*i*_‖*ID*_*i*_‖*α*_*i*_‖*r*_*i*_)*mod* *n*_0_, and writes {*B*_1_, *B*_2_, *β*_*i*_} to the smart card *SC*_*i*_.
4. Authentication and Key Agreement
  After node and user registration is complete, the user, *GWN*, and node can start authentication and key agreement. Figures 2 and 3 shows the authentication and key agreement phase.
  Step A1: User *U*_*i*_ inputs *ID*_*i*_, *PW*_*i*_, *BIO*_*i*_, smart card *SC*_*i*_ calculates *α*_i_^*^ = *Rep*(*BIO*_*i*_, *β*_*i*_), *r*_i_^*^ = *B*_1_ ⊕ *h*(*ID*_*i*_‖*α*_i_^*^‖*PW*_*i*_), *HPW*_i_^*^ = *h*(*PW*_*i*_‖*α*_i_^*^), *B*_2_^*^ = *h*(*HPW*_i_^*^‖*ID*_*i*_‖*α*_i_^*^‖*r*_i_^*^)*mod* *n*_0_, *SC*_*i*_ verifies whether *B*_2_^*^ is equal to *B*_2_ and continues it is; otherwise, terminate. User *U*_*i*_ chooses a random number *r*_*u*_ ∈ *Z*_p_^*^ and calculates *R*_*u*_ = *r*_*u*_·*P*, *R*_*ig*_ = *r*_*i*_·*R*_*g*_, *K*_*i*_ = *h*(*TID*_*i*_‖*HPW*_*i*_), *x*_*i*_ = *e*_*i*_ ⊕ *R*_*ig*_ ⊕ *K*_*i*_, *TID*_*i*_^′^ = *h*(*ID*_*i*_‖*α*_*i*_‖*r*_*u*_), *C*_*u*_ = *h*(*R*_*u*_‖*x*_*i*_^′^), *D*_0_ = *r*_*u*_·*R*_*g*_, *D*_1_ = *h*(*D*_*0*_‖*TID*_*i*_‖*HPW*_*i*_), *D*_2_ = *TID*_*i*_^′^ ⊕ (*D*_1_‖*x*_*i*_), choose time *T*_1_, calculate *D*_3_ = *h*(*TID*_*i*_^′^‖*D*_*0*_‖*x*_*i*_‖*K*_*i*_‖*T*_1_). *U*_*i*_ sends {*R*_*u*_, *D*_*2*_, *D*_*3*_, *TID*_*i*_, *T*_1_} to the *GWN*.
  Step A2: The gateway *GWN* receives the message and selects *T*_2_, verifies whether *|T*_2_ − *T*_1_*|* is less than or equal to △*T* and continues if it is, otherwise terminates. The *GWN* calculates *D*_0_^*^ = *r*_*g*_·*R*_*u*_, *x*_*i*_ = *h*(*TID*_*i*_‖*K*_*G*_), *D*_1_^*^ = *h*(*D*_0_^*^‖*TID*_*i*_‖*HPW*_*i*_), *TID*_*i*_^′*^ = *D*_2_ ⊕ (*D*_1_^*^‖*x*_*i*_), *K*_*i*_ = *h*(*TID*_*i*_‖*HPW*_*i*_), *D*_3_^*^ = *h*(*TID*_*i*_^′*^‖*D*_0_^*^‖*x*_*i*_‖*K*_*i*_‖*T*_1_), verifies whether *D*_3_^*^ is equal to *D*_3_ and continues if it is, *List* plus one; otherwise, it is terminated. *GWN* calculates *x*_*i*_^′*^ = *h*(*TID*_*i*_^′*^‖*K*_*G*_), *C*_u_^*^ = *h*(*R*_*u*_‖*x*_*i*_^′*^), *D*_*4*_ = *r*_*g*_* ⊕ h*(*SID*_*j*_‖*x*_*j*_‖*T*_2_), *D*_*5*_ = *C*_*u*_ ⊕ *h*(*r*_*g*_‖*x*_*j*_), *D*_*6*_ = *TID*_*i*_^′^ ⊕ *h*(*SID*_*j*_‖*r*_*g*_), *D*_7_ = *h*(*TID*_*i*_^′^‖*SID*_*j*_‖*C*_*u*_‖*r*_*g*_‖*x*_*j*_‖*T*_2_), and the *GWN* sends {*R*_*u*_, *R*_*g*_, *D*_4_, *D*_5_, *D*_6_, *D*_7_, *T*_2_} to *S*_*j*_.
  Step A3: The sensor *S*_*j*_ receives the message and selects *T*_*3*_, verifies whether *|T*_3_ − *T*_2_*|* is less than or equal to △*T* and continues it is; otherwise, it is terminated. *S*_*j*_ selects a random number *r*_*s*_ ∈ *Z*_p_^*^, calculates *R*_*s*_ = *r*_*s*_·*P*, *r*_g_^*^ = *D*_4_ ⊕ *h*(*SID*_*j*_‖*x*_*j*_‖*T*_2_), *C*_u_^*^ = *D*_5_ ⊕ *h*(*r*_g_^*^‖*x*_*j*_), *TID*_*i*_^’*^ = *D*_6_ ⊕ *h*(*SID*_*j*_‖*r*_g_^*^), *D*_7_^*^ = *h*(*TID*_*i*_^′*^‖*SID*_*j*_‖*C*_u_^*^‖*r*_g_^*^‖*x*_*j*_‖*T*_2_), verifies whether *D*_7_^*^ is equal to *D*_7_ and continues if it is; otherwise, it is terminated. *C*_*s*_ = *h*(*R*_*s*_‖*x*_*j*_), *R*_*su*_ = *r*_*s*_·*R*_*u*_, *SK*_*s*_ = *h*(*SID*_*j*_‖*r*_*g*_‖*R*_*su*_‖*C*_*u*_‖*C*_*s*_‖*TID*_*i*_^′^), *D*_*8*_ = *r*_*s*_·*R*_*g*_, *D*_*9*_ = *h*(*SID*_*j*_‖*r*_*g*_‖*D*_*8*_‖*x*_*j*_‖*C*_*s*_‖*T*_3_), *D*_10_ = *h*(*SID*_*j*_‖*SK*_*s*_‖*r*_*g*_*‖TID*_*i*_^′^) is calculated, and *S*_*j*_ sends {*R*_*s*_, *D*_9_, *D*_10_, *T*_3_} to the *GWN*.
  Step A4: The gateway *GWN* receives the message and selects *T*_4_, verifies whether *|T*_4_ − *T*_3_*|* is less than or equal to △*T* and continues if it is; otherwise, it is terminated. The *GWN* calculates *C*_s_^*^ = *h*(*R*_*s*_‖*x*_*j*_), *D*_8_^*^ = *r*_*g*_·*R*_*s*_, *D*_9_^*^ = *h*(*SID*_*j*_‖*r*_*g*_‖*D*_8_^*^‖*x*_*j*_‖*C*_s_^*^‖*T*_*3*_), verifies whether *D*_9_^*^ is equal to *D*_*9*_ and continues if it is; otherwise, it is terminated. *D*_11_ = *r*_*g*_ ⊕ *h*(*D*_*0*_‖*x*_*i*_^′^‖*T*_4_), *D*_12_ = *C*_*s*_ ⊕ *h*(*x*_*i*_^′^‖*r*_*g*_), *D*_*13*_ = *SID*_*j*_ ⊕ *h*(*D*_12_‖*x*_*i*_^′^‖*r*_*g*_), *K*_*i*_^′^ = *h*(*TID*_*i*_^′^‖*HPW*_*i*_), *e*_*i*_^′^ = *x*_*i*_^′^ ⊕ *R*_*ug*_ ⊕ *K*_*i*_^′^, *D*_*14*_ = *h*(*TID*_*i*_^′^‖*x*_*i*_^′^‖*K*_*i*_^′^‖*r*_*g*_‖*C*_*s*_‖*SID*_*j*_‖*D*_0_‖*T*_4_) is calculated and *{TID*_*i*_^′^, *K*_*i*_^′^, *List}* is updated, and the *GWN* sends {*R*_*s*_, *e*_*i*_^′^, *D*_10_, *D*_11_, *D*_12_, *D*_13_, *D*_14_, *T*_4_} to *U*_*i*_.
  Step A5: User *U*_*i*_ receives the message and selects *T*_5_, verifies whether *|T*_5_ − *T*_4_*|* is less than or equal to △*T* and continues it is; otherwise, it is terminated. *U*_*i*_ calculates *K*_*i*_^′^ = *h*(*TID*_*i*_^′^‖*HPW*_*i*_), *x*_*i*_^′*^ = *e*_*i*_^′^ ⊕ *R*_*ug*_ ⊕ *K*_*i*_^′^, *C*_u_^*^ = *h*(*R*_*u*_‖*x*_*i*_^′*^), *r*_g_^*^ = *D*_11_ ⊕ *h*(*D*_*0*_‖*x*_*i*_^′*^‖*T*_4_), *C*_s_^*^ = *D*_12_ ⊕ *h*(*x*_*i*_^′*^‖*r*_g_^*^), *SID*_j_^*^ = *D*_13_ ⊕ *h*(*D*_12_‖*x*_*i*_^′*^‖*r*_g_^*^), *D*_14_^*^ = *h*(*TID*_*i*_^′^‖*x*_*i*_^′*^‖*K*_*i*_^′^‖*r*_g_^*^‖*C*_s_^*^‖*SID*_j_^*^‖*D*_0_‖*T*_4_), verifies whether *D*_14_^*^ is equal to *D*_14_ and continues if equal; otherwise, it is terminated. *R*_*us*_ = *r*_*u*_·*R*_*s*_, *SK*_*u*_ = *h*(*SID*_*j*_‖*r*_*g*_‖*R*_*us*_‖*C*_*u*_‖*C*_*s*_‖*TID*_*i*_^′^), *D*_10_^*^ = *h*(*SID*_*j*_‖*SK*_*u*_‖*r*_*g*_‖*TID*_*i*_^*′*^*)* is calculated, whether *D*_10_^*^ is equal to *D*_10_ is verified, and it continues if it is; otherwise, it is terminated. This completes the two-way authentication and negotiates the session key *SK* for user *U*_*i*_ and sensor *S*_*j*_. Finally, *U*_*i*_ calculates *B*_1_^′^ = *h*(*ID*_*i*_‖*α*_*i*_‖*PW*_*i*_) ⊕ *r*_*u*_, *B*_*2*_^′^ = *h*(*HPW*_*i*_‖*ID*_*i*_‖*α*_*i*_‖*r*_*u*_)*mod* *n*_0_ with *B*_*1*_^′^, *B*_*2*_^′^, *e*_*i*_^′^ replacing *B*_1_, *B*_2_, *e*_*i*_ within the smart card *SC*_*i*_.
5. Password Update.
  Users can also perform a password update at any time after completing the authentication and key agreement. The password update process is shown in Fig. 4.
  Step P1: User *U*_*i*_ inputs *ID*_*i*_, *PW*_*i*_, *BIO*_*i*_, smart card *SC*_*i*_ calculates *α*_i_^*^ = *Rep*(*BIO*_*i*_*,β*_*i*_), *r*_u_^*^ = *B*_*1*_* ⊕ h*(*ID*_*i*_‖*α*_i_^*^‖*PW*_*i*_), *HPW*_i_^*^ = *h*(*PW*_*i*_‖*α*_i_^*^), *B*_2_^*^ = *h*(*HPW*_i_^*^‖*ID*_i_^*^‖*α*_i_^*^‖*r*_u_^*^)*mod* *n*_0_, verifies whether *B*_2_^*^ is equal to *B*_2_ and continues if it is; otherwise, it is terminated. *SC*_*i*_ calculates *TID*_*i*_ = *h*(*ID*_*i*_‖*α*_*i*_‖*r*_*u*_), *K*_*i*_ = *h*(*TID*_*i*_‖*HPW*_*i*_), *R*_*ug*_ = *r*_*u*_·*R*_*g*_, *x*_*i*_ = *e*_*i*_ ⊕ *R*_*ug*_ ⊕ *K*_*i*_.
  Step P2: User *U*_*i*_ enters the new password *PW*_*i*_^*new*^, smart card *SC*_*i*_ calculates *HPW*_*i*_^*new*^ = *h*(*PW*_*i*_^*new*^‖*α*_*i*_), *K*_*i*_^*new*^ = *h*(*TID*_*i*_‖*HPW*_*i*_^*new*^), *e*_*i*_^*new*^ = *R*_*ug*_ ⊕ *K*_*i*_^*new*^ ⊕ *x*_*i*_, *B*_*1*_^*new*^ = *h*(*ID*_*i*_‖*α*_*i*_‖*PW*_*i*_^*new*^) ⊕ *r*_*u*_, *B*_*2*_^*new*^ = *h*(*HPW*_*i*_^*new*^‖*ID*_*i*_‖*α*_*i*_‖*r*_*u*_)*mod* *n*_0_, replacing *B*_1_*,B*_2_*,e*_*i*_ in smart card *SC*_*i*_ with *B*_1_^*new*^, *B*_*2*_^*new*^,* e*_*i*_^*new*^, and the password update is completed.
6. Smart Card Logout
  Smart Card Logout can be performed when the user's Smart Card is no longer in use. The smart card logout process is shown in Fig. 5.
  Step S1: User *U*_*i*_ inputs *ID*_*i*_, *PW*_*i*_, *BIO*_*i*_, calculates *α*_i_^*^ = *Rep*(*BIO*_*i*_*,β*_*i*_), *r*_u_^*^ = *B*_*1*_* ⊕ h*(*ID*_*i*_‖*α*_i_^*^‖*PW*_*i*_), *HPW*_i_^*^ = *h*(*PW*_*i*_‖*α*_i_^*^), *B*_2_^*^ = *h*(*HPW*_i_^*^‖*ID*_*i*_‖*α*_i_^*^‖*r*_u_^*^)*mod* *n*_0_, verifies whether *B*_2_^*^ is equal to *B*_*2*_ and continues if it is; otherwise, it is terminated. *K*_*i*_ = *h*(*TID*_*i*_‖*HPW*_*i*_), *R*_*ug*_ = *r*_*u*_·*R*_*g*_, *x*_*i*_ = *e*_*i*_ ⊕ *R*_*ug*_ ⊕ *K*_*i*_ is calculated, time *T*_1_ is chosen, *L*_*o*_ = *x*_*i*_ ⊕ *h*(*K*_*i*_‖*T*_*1*_) is calculated, and *U*_*i*_ sends {*TID*_*i*_, *L*_*o*_, *T*_1_} to the *GWN*.
  Step S2: The gateway *GWN* receives the message and selects *T*_*2*_, verifies whether |*T*_2_ − *T*_1_| is less than or equal to △*T* and continues if it is; otherwise, it is terminated. The *GWN* calculates *K*_*i*_^*′*^ = *h*(*TID*_*i*_‖*HPW*_*i*_), *x*_i_^*^ = *L*_*o*_ ⊕ *h*(*K*_*i*_^*′*^‖*T*_1_), *x*_*i*_ = *h*(*TID*_*i*_‖*K*_*G*_), verifies whether *x*_i_^*^ is equal to *x*_*i*_ and continues if it is; otherwise, it is terminated. Finally, the messages associated with *U*_*i*_{*TID*_*i*_, *HPW*_*i*_, *List*} are deleted, and smart card revocation is completed.

## Security analysis

This section provides a formal security analysis of the scheme using BAN logic. The informal security analysis is performed through Propositions 1 to 11 for a variety of known attacks. The security analysis proves the correctness of the scheme; it can resist various security attacks and has high security characteristics^28^.

### Formal analysis based on BAN logic

Next, BAN logic is used to demonstrate the security of the scheme.

1. Goals
  *G*1: $$S_{j} | \equiv U_{i} \mathop \leftrightarrow \limits^{SK} S_{j}$$ *G*2: $$S_{j} \left| { \equiv U_{i} } \right| \equiv U_{i} \mathop \leftrightarrow \limits^{SK} S_{j}$$
  *G*3: $$U_{i} | \equiv S_{j} \mathop \leftrightarrow \limits^{SK} U_{i}$$ *G*4: $$U_{i} \left| { \equiv S_{j} } \right| \equiv S_{j} \mathop \leftrightarrow \limits^{SK} U_{i}$$
2. Idealized Forms
  *M*1: $$U_{i} \to GWN:R_{u} ,D_{2} ,T_{1} ,TID_{i} , < TID_{i}^{{\prime }} ,D_{0} ,k_{i} >_{{x_{i} }}$$
  *M*2: $$GWN \to S_{j} :R_{u} ,R_{g} ,D_{4} ,D_{5} ,D_{6} ,T_{2} , < TID_{i}^{{\prime }} ,U_{i} | \equiv C_{u} ,r_{g} >_{{x_{j} }}$$
  *M*3: $$S_{j} \to GWN:R_{s} ,D_{10} ,T_{3} , < D_{8} ,r_{g} ,S_{j} | \equiv C_{s} >_{{x_{j} }}$$
  *M*4: $$GWN \to U_{i} :e_{i}^{{\prime }} ,R_{s} ,D_{10} ,D_{11} ,D_{12} ,D_{13} ,T_{4} , < TID_{i}^{{\prime }} ,x_{i}^{{\prime }} ,D_{0} ,r_{g} ,S_{j} | \equiv C_{s} >_{{k_{i}^{{\prime }} }}$$
3. Assumptions
  *A*1: $$GWN| \equiv U_{i} \mathop \leftrightarrow \limits^{{x_{i} }} GWN$$ *A*2: $$S_{j} | \equiv GWN\mathop \leftrightarrow \limits^{{x_{j} }} S_{j}$$
  *A*3: $$GWN| \equiv S_{j} \mathop \leftrightarrow \limits^{{x_{j} }} GWN$$ *A*4: $$U_{i} | \equiv GWN\mathop \leftrightarrow \limits^{{k_{i}^{{\prime }} }} U_{i}$$
  *A*5: $$GWN| \equiv \# \left( {C_{u} } \right)$$ *A*6: $$S_{j} | \equiv \# \left( {r_{g} } \right)$$
  *A*7: $$GWN| \equiv \# \left( {C_{s} } \right)$$ *A*8: $$U_{i} | \equiv \# \left( {r_{g} } \right)$$
  *A*9: $$GWN\left| { \equiv U_{i} } \right| \Rightarrow < D_{3} >$$ *A*10: $$S_{j} \left| { \equiv GWN} \right| \Rightarrow < D_{7} >$$
  *A*11: $$GWN\left| { \equiv S_{j} } \right| \Rightarrow < D_{9} >$$ *A*12: $$U_{i} \left| { \equiv GWN} \right| \Rightarrow < D_{14} >$$
  *A*13: $$S_{j} | \equiv \# (C_{u} )$$ *A*14: $$U_{i} | \equiv \# (C_{s} )$$
  *A*15: $$S_{j} \left| { \equiv U_{i} } \right|\sim U_{i} \mathop \leftrightarrow \limits^{SK} S_{j}$$ *A*16: $$U_{i} \left| { \equiv S_{j} } \right|{\sim }U_{i} \mathop \leftrightarrow \limits^{SK} S_{j}$$
4. Main Proofs
  From *M1*, they can get *S1:* $GWN \triangleleft < D_{3} >_{{x_{i} }}$.
  From *S1*, *A1*, *R1*, they can get *S2*: $GWN\left| { \equiv U_{i} } \right|\sim < D_{3} >$.
  From *A5*, *R4*, they can get *S3*: $GWN| \equiv \# ( < D_{3} > )$.
  From *S2*, *S3*, *R2*, they can get *S4*: $GWN\left| { \equiv U_{i} } \right| \equiv < D_{3} >$.
  From *S4*, *A9*, *R3*, they can get *S5*: $GWN| \equiv < D_{3} >$.
  From *M2*, they can get *S6*: $S_{j} \triangleleft < D_{7} >_{{x_{j} }}$.
  From *S6*, *A2*, *R1*, they can get *S7*: $S_{j} \left| { \equiv GWN} \right|{\sim } < D_{7} >$.
  From *A6*, *R4*, they can get *S8*: $S_{j} | \equiv \# ( < D_{7} > )$.
  From *S7*, *S8*, *R2*, they can get *S9*: $S_{j} \left| { \equiv GWN} \right| \equiv < D_{7} >$.
  From *S9*, *A10*, *R3*, they can get *S10*: $S_{j} | \equiv < D_{7} >$.
  From *S10*, *R5*, they can get *S11*: $S_{j} \left| { \equiv U_{i} } \right| \equiv C_{u}$.
  $$SK = h\left( {SID_{j} ||r_{g} ||R_{su} ||C_{u} ||C_{s} ||TID_{i}^{{\prime }} } \right).$$
  From *S11*, *A13*, *SK*, *R6*, they can get *S12*: $S_{j} | \equiv U_{i} \mathop \leftrightarrow \limits^{SK} S_{j}$, they have achieved *G*1.
  From *S12*, *A13*, *A15*, *R2*, *R4*, they can get S13: $S_{j} \left| { \equiv U_{i} } \right| \equiv U_{i} \mathop \leftrightarrow \limits^{SK} S_{j}$, they have achieved *G*2.
  From *M3*, they can get *S14:* $GWN \triangleleft < D_{9} >_{{x_{j} }}$.
  From *S14*, *A3*, *R1*, they can get *S15*: $GWN\left| { \equiv S_{j} } \right|{\sim } < D_{9} >$.
  From *A7*, *R4*, they can get *S16*: $GWN| \equiv \# ( < D_{9} > )$.
  From *S15*, *S16*, *R2*, they can get *S17*: $GWN\left| { \equiv S_{j} } \right| \equiv < D_{9} >$.
  From *S17*, *A11*, *R3*, they can get *S18*: $GWN| \equiv < D_{9} >$.
  From *M4*, they can get *S19:* $U_{i} \triangleleft < D_{14} >_{{k_{i}^{{\prime }} }}$.
  From *S19*, *A4*, *R1*, they can get *S20*: $U_{i} \left| { \equiv GWN} \right|{\sim } < D_{14} >$.
  From *A8*, *R4*, they can get *S21*: $U_{i} | \equiv \# ( < D_{14} > )$.
  From *S20*, *S21*, *R2*, they can get *S22*: $U_{i} \left| { \equiv GWN} \right| \equiv < D_{14} >$.
  From *S22*, *A12*, *R3*, they can get *S23*: $U_{i} | \equiv < D_{14} >$.
  From *S23*, *R5*, they can get *S24*: $U_{i} \left| { \equiv S_{j} } \right| \equiv C_{s}$.
  $$SK = h\left( {SID_{j} ||r_{g} ||R_{us} ||C_{u} ||C_{s} ||TID_{i}^{{\prime }} } \right).$$
  From *S24*, *A14*, *SK*, *R6*, they can get *S25*: ${U}_{i}|\equiv {S}_{j}\stackrel{SK}{\leftrightarrow }{U}_{i}$, they have achieved *G*3.
  From *S25*, *A14*, *A16*, *R2*, *R4*, they can get S26: ${U}_{i} |\equiv {S}_{j}|\equiv {S}_{j}\stackrel{SK}{\leftrightarrow }{U}_{i}$, they have achieved *G*4.

In summary, according to the BAN logic rules, the security objectives *G1* to *G4* of this scheme have been achieved, and the security of the scheme has been proven.

### Formal analysis based on the random oracle model

#### Theorem 1

In a scenario where an adversary attacker (*A*) operates within probabilistic polynomial time (PPT) against a protocol (*P*) in a random oracle, *A* is allowed to make up to *q*_*s*_ Send ($\mathop \prod \limits_{I}^{*} , m$) queries, *q*_*e*_ Execute ($\mathop \prod \limits_{U}^{i} , \mathop \prod \limits_{GWN}^{k} , \mathop \prod \limits_{S}^{j}$) queries, and *q*_*h*_ oracle queries. Let *D* denote the password space, which follows a Zipf distribution with parameters *C*^′^ and *s*^′^^16^. Additionally, *l* represents the output length of the hash function and AKE represents authenticated key agreement. In the context of the random oracle model, the probability *P* of *A* successfully compromising the protocol in PPT is defined as follows:

30 $${\text{Adv}}_{{\text{P}}}^{{{\text{AKE}}}} \left( {\text{A}} \right) = 2\left| {{\text{Pr}}\left[ {{\text{S}}_{4} \left] { - {\text{Pr}}} \right[{\text{S}}_{0} } \right]} \right| \le {\text{max}}\left\{ {\frac{{{\text{q}}_{{\text{s}}} }}{{2^{{{\text{l}}_{{\upalpha }} - 1}} }},2{\text{C}}^{{\prime }} {\text{q}}_{{\text{s}}}^{{{\text{s}}{\prime }}} ,\frac{{{\text{q}}_{{\text{s}}} }}{{2^{{{\text{l}} - 1}} }}} \right\} + \frac{{{\text{q}}_{{\text{s}}} }}{{2^{{{\text{l}} - 1}} }} + \frac{{{\text{q}}_{{\text{h}}}^{2} }}{{2^{{\text{l}}} }} + \frac{{\left( {{\text{q}}_{{\text{s}}} + {\text{q}}_{{\text{e}}} } \right)^{2} }}{{{\text{p}} - 1}}$$

Proof: The scheme is divided into five games, labelled *G*_*i*_(*i* = 1, 2, 3, 4, 5). In each game, there is a condition denoted as *S*_*i*_, indicating that *A* successfully predicts a bit *b* before advancing in the game.

*G*_0_: It mimics a real attack in the random oracle model, where *A* has full access to all oracles. Hence,

31 $${{\text{Adv}}}_{{\text{P}}}^{{\text{AKE}}}({\text{A}})=2{\text{Pr}}[{{\text{S}}}_{0}]-1$$

*G*_1_: In *G*_1_, *A* conducts a passive attack, intercepting messages through the Excute(*) query and attempting to guess the output of the Test (${\prod }_{S}^{j})$ query. However, the impossibility of deducing *SK* = *h*(*SID*_*j*_‖*r*_*g*_‖*R*_*us*_‖*C*_*u*_‖*C*_*s*_‖*TID*_*i*_^′^) means that *A*'s advantage in a successful attack does not increase. Hence,

32 $${\text{Pr}}[{{\text{S}}}_{1}]={\text{Pr}}[{{\text{S}}}_{0}]$$

*G*_2_: *A* is allowed to make Send (${\prod }_{I}^{*}, m$) and *H* queries to persuade the legitimate communicator with forged messages. The simulation concludes only if *A* manages to discover collisions and successfully constructs convincing messages. The probabilities of their occurrence, based on the birthday paradox^29^, are (${q}_{h}^{2}$/${2}^{l+1}$) and ((*q*_*s*_ + *q*_*e*_)^2^/2(*p*-1)). Hence,

33 $$|{\text{Pr}}[{{\text{S}}}_{2}]-{\text{Pr}}[{{\text{S}}}_{1}]|\le \frac{{{\text{q}}}_{{\text{h}}}^{2}}{{2}^{{\text{l}}+1}}+\frac{{({{\text{q}}}_{{\text{s}}}+{{\text{q}}}_{{\text{e}}})}^{2}}{2({\text{p}}-1)}$$

*G*_3_: This game is distinct from the earlier games because if *A* successfully guesses the correct authentication Factors *D*_3_*, D*_7_*, D*_9_*,* and *D*_14_*.* The simulation concludes if *H* queries are not utilized. It is identical to the previous games in all aspects, except for situations where correct authentication is refused. Hence,

34 $$|{\text{Pr}}[{{\text{S}}}_{3}]-{\text{Pr}}[{{\text{S}}}_{2}]|\le \frac{{{\text{q}}}_{{\text{s}}}}{{2}^{{\text{l}}}}$$

*G*_4_: In this game, *A* can acquire more information through the Corrupt (${\prod }_{U}^{i}, a$) query. *A* successfully guesses *α*_*i*_ with a length of *l*_α_, with a probability of (*q*_*s*_/2^*l*^_*α*_). Additionally, *A* successfully guesses the victim's password with a probability of *C*^′^$q_{s}^{{s{\prime }}}$. The likelihood of *A* guessing the correct *x*_*i*_ is (*q*_*s*_/2^*l*^). Hence,

35 $$|{\text{Pr}}[{{\text{S}}}_{4}]-{\text{Pr}}[{{\text{S}}}_{3}]|\le {\text{max}}\left\{\frac{{{\text{q}}}_{{\text{s}}}}{{2}^{{{\text{l}}}_{\mathrm{\alpha }}}},\mathrm{C{\prime}}{{\text{q}}}_{{\text{s}}}^{\mathrm{s{\prime}}},\frac{{{\text{q}}}_{{\text{s}}}}{{2}^{{\text{l}}}}\right\}$$

36 $${\text{Pr}}[{{\text{S}}}_{4}]=\frac{1}{2}$$

Based on Eqs. (31) to (36), they can infer either Conclusion (30) or Conclusion (37):

37 $${\text{Adv}}_{{\text{P}}}^{{{\text{AKE}}}} \left( {\text{A}} \right) = 2\left| {{\text{Pr}}\left[ {{\text{S}}_{4} \left] { - {\text{Pr}}} \right[{\text{S}}_{0} } \right]} \right| \le {\text{max}}\left\{ {\frac{{{\text{q}}_{{\text{s}}} }}{{2^{{{\text{l}}_{{\upalpha }} - 1}} }},2{\text{C}}^{{\prime }} {\text{q}}_{{\text{s}}}^{{{\text{s}}{\prime }}} ,\frac{{{\text{q}}_{{\text{s}}} }}{{2^{{{\text{l}} - 1}} }}} \right\} + \frac{{{\text{q}}_{{\text{s}}} }}{{2^{{{\text{l}} - 1}} }} + \frac{{{\text{q}}_{{\text{h}}}^{2} }}{{2^{{\text{l}}} }} + \frac{{\left( {{\text{q}}_{{\text{s}}} + {\text{q}}_{{\text{e}}} } \right)^{2} }}{{{\text{p}} - 1}}$$

### Formal security verification via ProVerif^30^

This section presents the formal security verification of the proposed scheme by using the Pi calculus-based simulation tool ProVerif. To date, ProVerif has been used to verify many protocols and demonstrate their correctness and robust properties, so ProVerif is used in this study to rectify the secrecy and authentication properties of the focal protocol.

**Figure 6:**
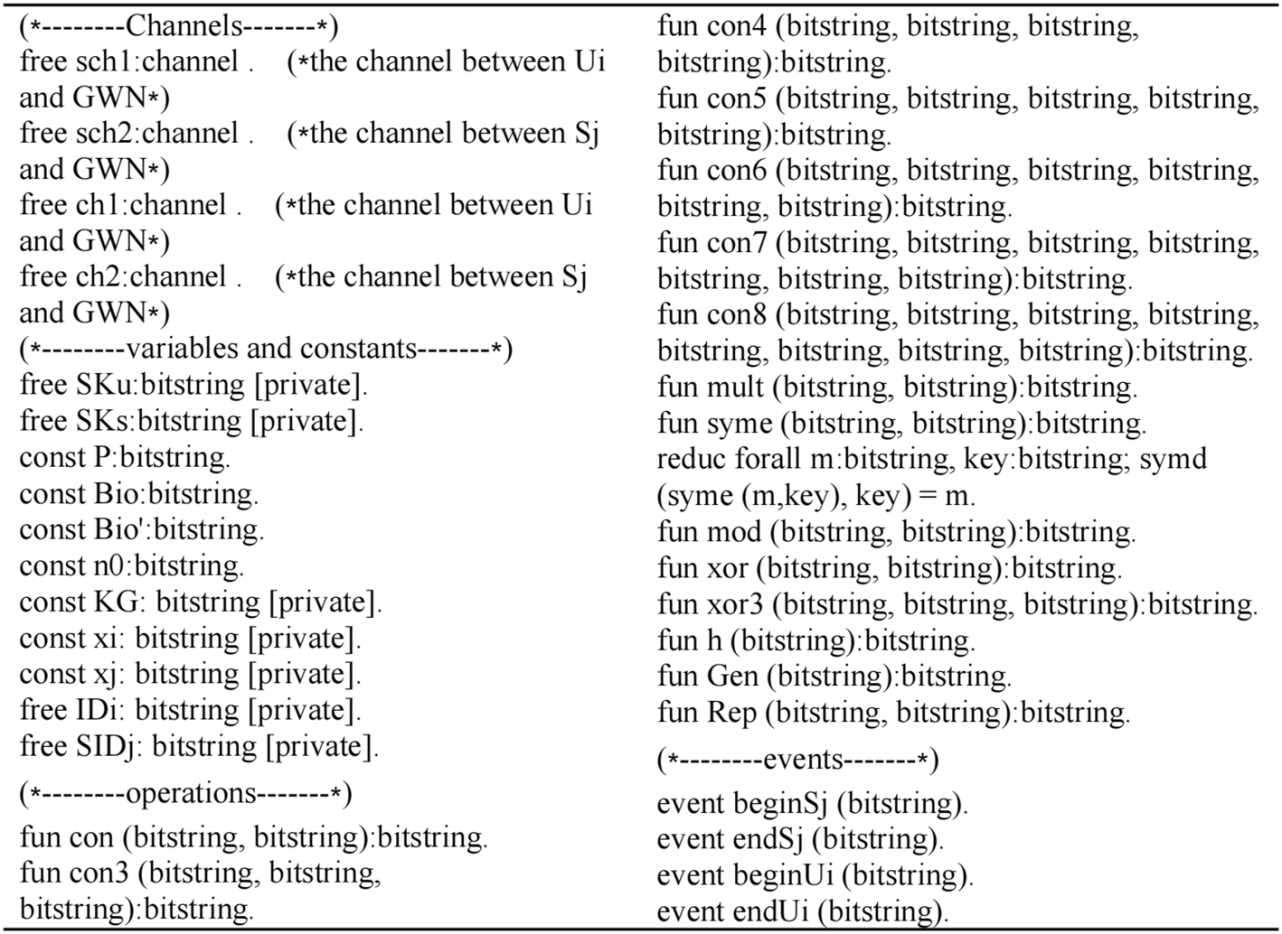
Define the channels, variables, constants, operations and events.

**Figure 7:**
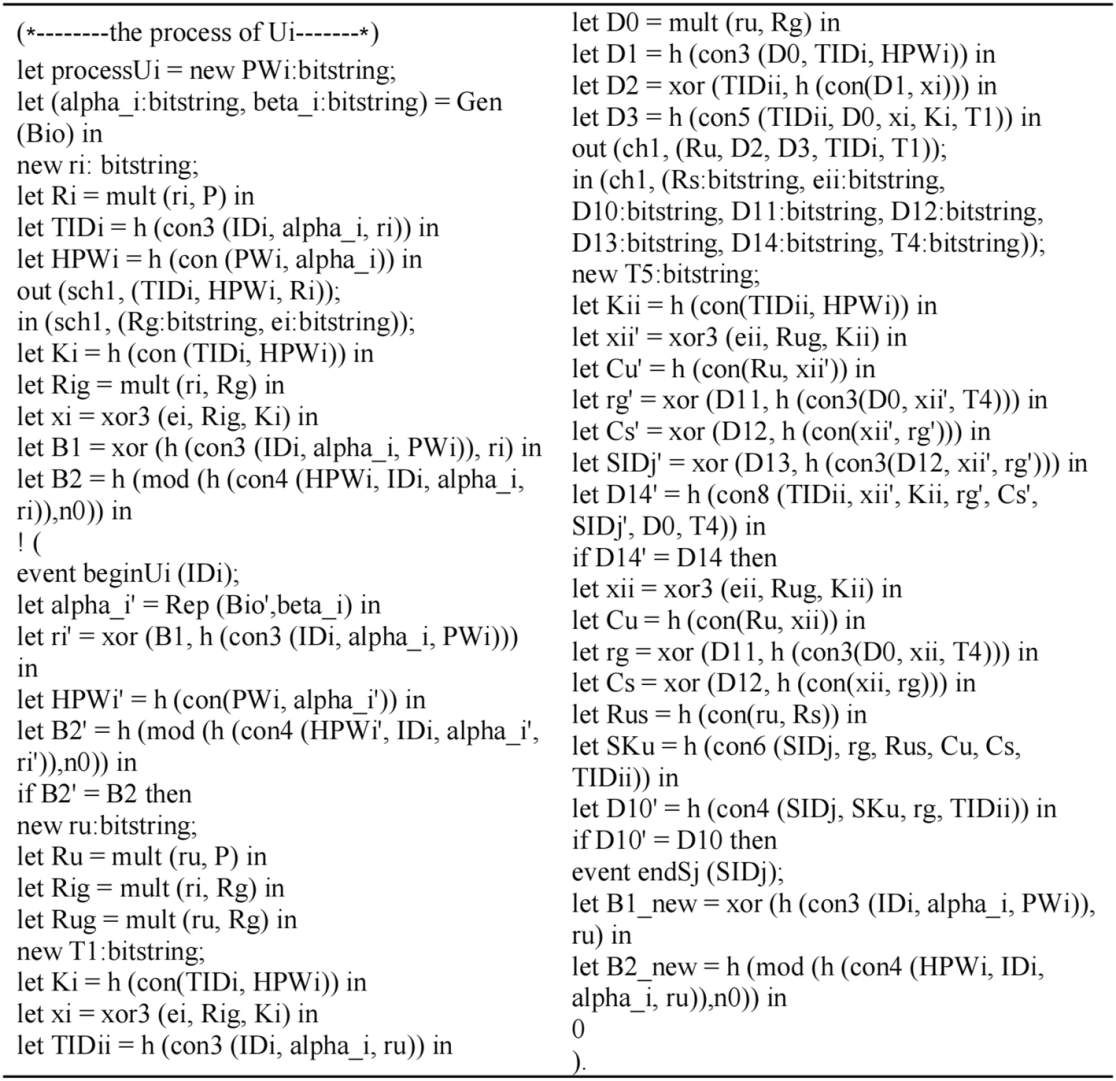
The process of *U*_i_.

**Figure 8:**
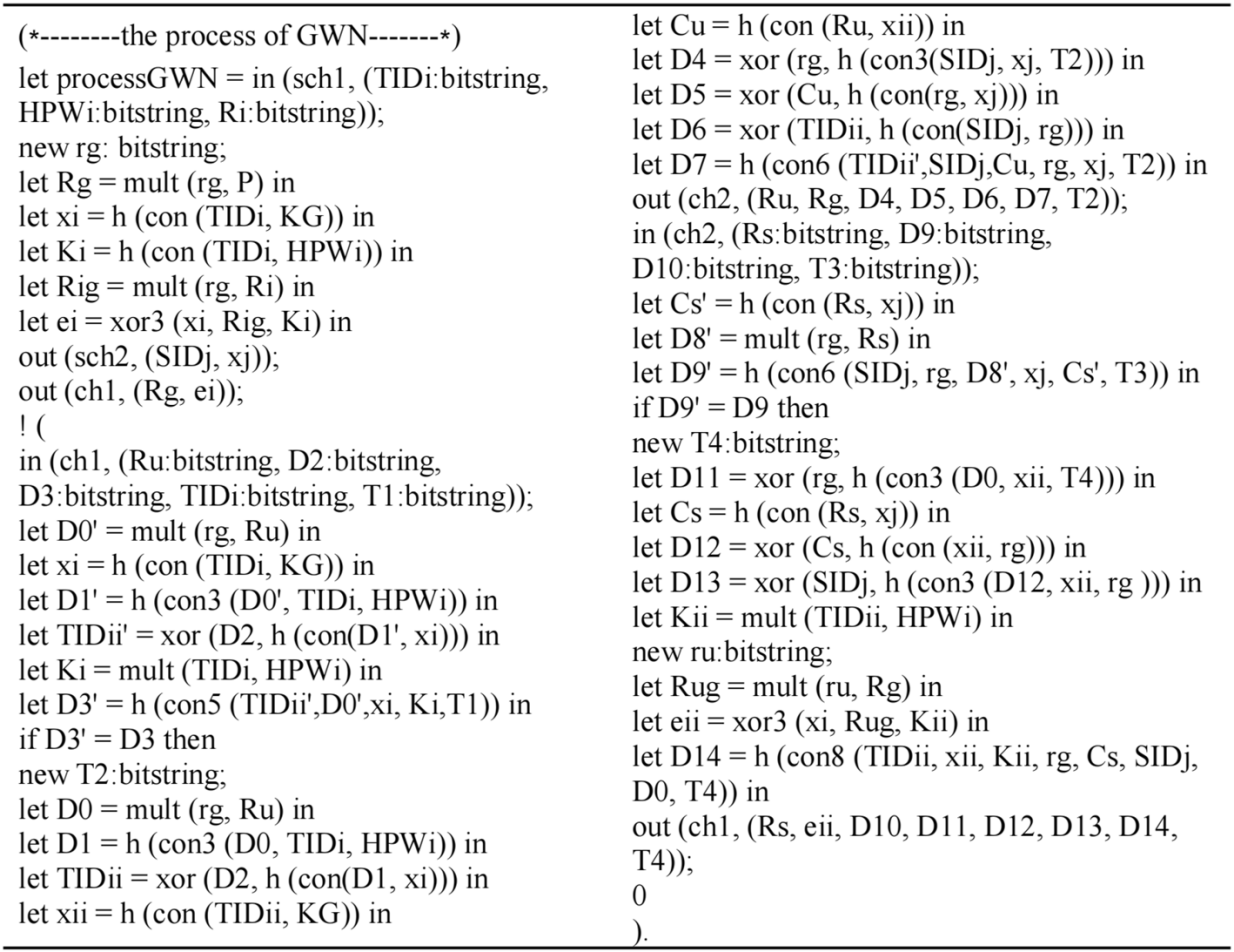
The process of *GWN.*

**Figure 9:**
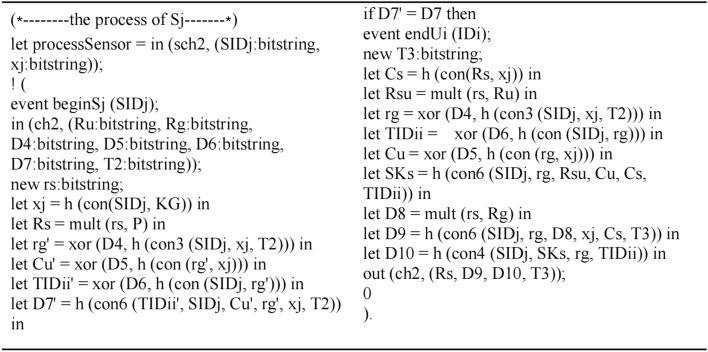
The process of *S*_*j*_.

**Figure 10:**
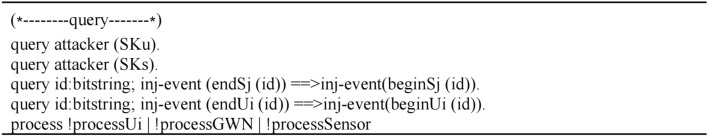
Define the queries and simulate the scheme.

**Figure 11:**
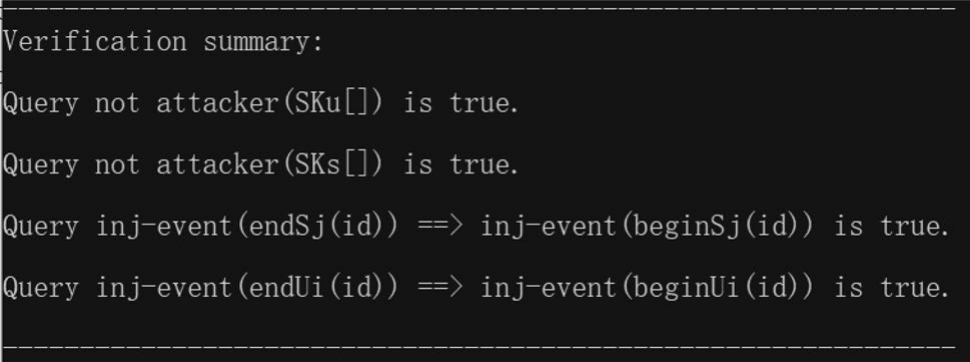
Outputs of the Proverif verification.

The channels, variables, constants, operations and events are defined as shown in Fig. 6:

According to the proposed scheme execution, they define the process of *U*_i_ as shown in Fig. 7:

The process of *GWN* is modeled as shown in Fig. 8:

The process of *S*_*j*_ is modeled as shown in Fig. 9:

The queries are defined and the whole scheme is simulated as executing in parallel as shown in Fig. 10:

The outputs of the ProVerif verification is shown in Fig. 11:

Results (1) and (2) indicate the secrecy of the proposed scheme because of the failing query attack on session keys *SK*_*S*_ and *SK*_*U*_. Moreover, Results (3) and (4) confirm the successful mutual authentication between *U*_*i*_ and *S*_*j*_. In other words, the proposed scheme not only provides the secrecy of the session key, but also achieves the authentication property by verifying the correspondence assertions in the Dolev-Yao model.

### Informal analysis

This scheme can resist many common attacks and effectively address the shortcomings of existing schemes. The proof of this is as follows:

#### Proposition 1

The scheme has anonymity.

#### Proof

All identity *ID* in the scheme are not transmitted in clear text in the public channel, and the identity identifiers *TID*_*i*_ = *h*(*ID*_*i*_‖*α*_*i*_‖*r*_*i*_) and *TID*_*i*_^′^ = *h*(*ID*_*i*_‖*α*_*i*_‖*r*_*u*_) are used to replace the *ID* for transmission^17^. Assuming that the attacker intercepts *TID*_*i*_, according to the one-way property of the hash function, the attacker cannot resolve *ID*_*i*_^31^. In addition, even if the attacker intercepts both *TID*_*i*_ and *TID*_*i*_^′^, it is impossible to determine whether the two parameters come from the same *ID*; hence, the scheme has anonymity.

#### Proposition 2

The scheme is resistant to registered legitimate user attacks.

#### Proof

Suppose attacker *U*_*a*_ registers legitimate user *ID*_*a*_ and calculates *TID*_*a*_ = *h*(*ID*_*a*_‖*α*_*a*_‖*r*_*a*_). *U*_*a*_ registers with gateway *GWN*, which calculates *x*_*a*_ = *h*(*TID*_*a*_‖*K*_*G*_), *K*_*a*_ = *h*(*TID*_*a*_‖*HPW*_*a*_). The *TID*_*a*_ generated by the attacker based on *ID*_*a*_ is different from the *TID*_*s*_ of other legitimate users, and the *x* and *K* generated by registering to GWN through *TID*_*a*_ are also different. Therefore, the scheme can resist registered legitimate user attacks by generating new identity information *TID*_*s*_, and the attacker cannot obtain messages to any other legitimate user by registering a legitimate user.

#### Proposition 3

The scheme is resistant to smart card loss attacks and offline guessing attacks^17^.

#### Proof

Suppose that a user’s smart card is lost or stolen, and the attacker obtains the card and the information it contains, *B*_1_ = *h*(*ID*_*i*_‖*α*_*i*_‖*PW*_*i*_) ⊕ *r*_*i*_, *B*_2_ = *h*(*HPW*_*i*_‖*ID*_*i*_‖*α*_*i*_‖*r*_*i*_)*mod* *n*_0_, by differential energy attack, because *B*_*1*_ and *B*_*2*_ are hash functions with one-way security. However, the attacker is unable to extract the password *PW*_*i*_ of user *U*_*i*_ from it. Second, if the attacker wishes to obtain the user's password *PW*_*i*_ through offline password guessing, he or she needs to have the biometric trait *α*_*i*_ and the private key *r*_*i*_, however, the attacker is not in possession of *α*_*i*_ and *r*_*i*_, and therefore, the attacker is unable to carry out an offline password guessing attack^32^. Again, *B*_2_ = *h*(*HPW*_*i*_‖*ID*_*i*_‖*α*_*i*_‖*r*_*i*_)*mod n*_0_, when *n*_0_ is taken large enough, the number of password guesses grows exponentially and it is not feasible to obtain the password by offline guessing. Finally, the gateway records the number of user authentication *List*, and it is impossible for an attacker to complete an offline guessing attack within a limited number of guesses. Therefore, the scheme resists smart card loss attacks and offline guessing attacks by means of hash functions, biometrics, modulo arithmetic, and recording the number of authentication times, which are infeasible regardless of whether the attacker tries to extract the password from the smart card or crack the password through offline guessing.

#### Proposition 4

The scheme is resistant to spoofed user attacks.

#### Proof

To disguise a user login gateway, the attacker needs to send {*R*_*u*_, *D*_2_, D_3_, *TID*_*i*_, *T*_1_} to the gateway, where *R*_*u*_ = *r*_*u*_·*P*, *TID*_*i*_^′^ = *h*(*ID*_*i*_‖*α*_*i*_‖*r*_*u*_), *C*_*u*_ = *h*(*R*_*u*_‖*x*_*i*_^′^), *D*_0_ = *r*_*u*_·*R*_*g*_, *D*_1_ = *h*(*D*_0_‖*TID*_*i*_‖*HPW*_*i*_), *D*_2_ = *TID*_*i*_^′^ ⊕ (*D*_1_‖*x*_*i*_), *D*_3_ = *h*(*TID*_*i*_^′^‖*D*_*0*_‖*C*_*u*_‖*x*_*i*_‖*K*_*i*_‖*T*_1_); the attacker needs to master the user’s private key *r*_*u*_, identifier *TID*_*i*_, password *PW*_*i*_, biometric *α*_*i*_, secret *x*_*i*_, key parameters *K*_*i*_, and so on, so it is clear that the attacker cannot master the above parameters at the same time and cannot make a spoofed user attack. Therefore, the scheme can resist spoofed user attacks by setting various parameters.

#### Proposition 5

The scheme is resistant to internal attacks.

#### Proof

There is a possibility that insiders leak user information at the gateway. In the user registration stage, the user’s registered password *PW*_*i*_ is protected by *HPW*_*i*_ = *h*(*PW*_*i*_‖*α*_*i*_), and the insider may obtain *HPW*_*i*_. Based on the unidirectional nature of the hash function, the insider is unable to compute *PW*_*i*_ by *HPW*_*i*_ = *h*(*PW*_*i*_‖*α*_*i*_)^33^. In addition, *HPW*_*i*_ also contains the user’s biometric *α*_*i*_, and the insider cannot obtain *α*_*i*_ to guess the correct *PW*_*i*_ by offline guessing. Therefore, the scheme can resist internal attacks by setting *HPW*_*i*_.

#### Proposition 6

The scheme is resistant to tampering attacks.

#### Proof

Suppose the attacker tampers with the message sent by the user to the gateway, and the gateway receives the message and needs to verify whether *D*_3_^*^ = *h*(*TID*_*i*_^′^‖*D*_0_^*^‖*C*_*u*_‖*x*_*i*_‖*K*_*i*_‖*T*_1_) is equal to *D*_3_. To crack *D*_3_, the attacker needs to have both the user's private key *r*_*u*_, identifier *ID*_*i*_, password *PW*_*i*_, secret *x*_*i*_, and key parameter *K*_*i*_^34^, etc. The above parameters are not propagated in plaintext over the public channel, and the attacker cannot verify them through the gateway. Therefore, the scheme makes it impossible for an attacker to authenticate *D*_*3*_ by setting multiple parameters. The scheme is resistant to tampering attacks.

#### Proposition 7

The scheme is resistant to replay attacks.

#### Proof

A replay attack occurs when an attacker sends a packet that has been received by the target for the purpose of spoofing the system. All the messages sent in the two-way authentication process contain the timestamp *T*, and all parties need to verify whether the time difference is less than △*T* after receiving the message. If the attacker carries out replay attacks, the replayed message can be recognized by verifying the timestamp. The scheme resists replay attacks by adding timestamps.

#### Proposition 8

The scheme is resistant to MITT attacks.

#### Proof

According to the challenge/response mechanism, both the user and the gateway or the sensor and the gateway need to verify each other’s identity. According to Propositions 4 and 6, which have already been proven, the attacker cannot disguise the user or tamper with the message, so the attacker cannot launch a MITT attack disguised as an intermediary. The same can be proven for the communication between sensors and gateways. In addition, timestamps and random numbers are fresh and cannot be forged by an MITT attack^35^. Therefore, an attacker cannot disguise him- or herself as an MITT to launch an attack. The scheme makes it impossible for the attacker to accomplish MITT attacks by authenticating the user, gateway, and sensor.

#### Proposition 9

The scheme is resistant to Denning-Sacco attacks^36^.

#### Proof

Suppose the attacker steals the agreement key *SK* = *h*(*SID*_*j*_‖*r*_*g*_‖*R*_*su*_‖*C*_*u*_‖*C*_*s*_‖*TID*_*i*_^′^). *SK* is the hash function’s hash value^37^, and according to its one-way property, the attacker cannot obtain the parameters in *SK*. In addition, the parameters in *SK* such as user private key *r*_*u*_, gateway private key *r*_*g*_, sensor private key *r*_*s*_, *C*_*u*_, and *C*_*s*_ are not transmitted in the public channel, and the attacker cannot complete the Denning-Sacco attack.Therefore, the scheme resists Denning-Sacco attacks by performing hash transformations on the session key *SK* and by making *SK* have more complex parameters.

#### Proposition 10

The scheme has forward security.

#### Proof

Assuming that the attacker intercepts the public keys *R*_*u*_ and *R*_*s*_ of the user and the sensor, the calculation of *SK* also requires *r*_*u*_, *r*_*g*_, *r*_*s*_, *C*_*u*_, and *C*_*s*_. None of these parameters are transmitted in the public channel, and they cannot be obtained by the attacker. An attacker trying to calculate *r*_*s*_ and *r*_*u*_ by *R*_*s*_ = *r*_s_*P and *R*_*u*_ = *r*_s_*P, or *r*_s_*R_*u*_ and *R*_s_*r_*u*_ by *R*_s_*R_*u*_ cannot do so because the above computations involve ECCDLP mathematical puzzles. Therefore, the scheme is forward-safe.

#### Proposition 11

The scheme enables both two-way authentication and key agreement.

#### Proof

The scheme through *D*_3_ = *h*(*TID*_*i*_^′^‖*D*_*0*_‖*C*_*u*_‖*x*_*i*_‖*K*_*i*_‖*T*_1_) and *D*_14_ = *h*(*TID*_*i*_^′^‖*x*_*i*_^′^‖*K*_*i*_^′^‖*r*_*g*_‖*C*_*s*_‖*SID*_*j*_‖*D*_*0*_‖*T*_4_) achieves two-way authentication of the user and the gateway and through *D*_7_ = *h*(*TID*_*i*_^′^‖*SID*_*j*_‖*C*_*u*_‖*r*_*g*_‖*x*_*j*_‖*T*_2_) and *D*_9_ = *h*(*SID*_*j*_‖*r*_*g*_‖*D*_*8*_‖*x*_*j*_‖*C*_*s*_‖*T*_3_) achieves two-way authentication of the gateway and the sensor, while the session key *SK*_*s*_ = *h*(*SID*_*j*_‖*r*_*g*_‖*R*_*su*_‖*C*_*u*_‖*C*_*s*_‖*TID*_*i*_^′^) = *h(SID*_*j*_‖*r*_*g*_‖*R*_*us*_‖*C*_*u*_‖*C*_*s*_‖*TID*_*i*_^′^) = *SK*_*u*_ is negotiated during the authentication process.

Table 3 shows the security comparison of each scheme. It can be seen that this scheme has better security.

**Table 3:** Comparison of security features.

	Xue et al.^16^	Mo et al.^39^	Deng et al.^40^	Meriam et al.^6^	Proposed Scheme
Year	2021	2022	2022	2022	2022
Forward security	×	×	×	×	√
Resist KSSTI attacks	×	×	×	√	√
Resist internal privilege attacks	√	×	√	×	√
Resist offline dictionary attacks	√	√	√	√	√
Clock synchronization	√	×	√	×	√
Anonymity	×	√	√	√	√
Resist MITM attacks	×	×	√	√	√
Resist user registration attacks	×	√	√	√	√

Remarks: √: Yes × : No.

## Efficiency analysis

The sensor nodes of WSNs have the characteristics of limited resources and low computation. In this section, they analyze the performance of scheme in analysed from two aspects—computation overhead and communication overhead—and the scheme is proven to be suitable for resource-constrained WSNs through comparisons with other schemes^38^.

### Computational overhead

The computational overhead is mainly considered for recovering biometric features, point multiplication, modular exponentiation, symmetric encryption/decryption, hashing, and so forth. The computational overhead of XOR and concatenation is very small and negligible compared to other operations. Referring to the literature^15^, the computational elapsed time is shown in Table 4; the comparison of computational overheads of each scheme is shown in Table 5.

**Table 4:** The notations, descriptions, and time consuming required for computational time.

Notations	Descriptions	Time consuming (ms)
*T*_*FE*_	Time of recover biometric features	1.989
*T*_*ecm*_	Time of point multiplication operation	1.989
*T*_*mm*_	Time of Modular exponentiation operation	0.171
*T*_*E/D*_	Time of symmetric encryption/decryption operations	0.00325
*T*_*h*_	Time of hash operation	0.0026

**Table 5:** Comparison of computational overhead.

	*U*_*i*_	*GWN*	*S*_*j*_	合计
Xue et al.^16^	13*T*_*h*_ + 1*T*_*FE*_	18*T*_*h*_	6*T*_*h*_	37*T*_*h*_ + 1*T*_*FE*_
Mo et al.^39^	2*T*_*ecm*_ + 12*T*_*h*_ + 1*T*_*FE*_	10*T*_*h*_ + 1*T*_*E/D*_	2*T*_*ecm*_ + 5*T*_*h*_ + 1*T*_*E/D*_	4*T*_*ecm*_ + 27*T*_*h*_ + 2*T*_*FE*_ + 1*T*_*FE*_
Deng et al.^40^	2*T*_*ecm*_ + 14*T*_*h*_ + 1*T*_*FE*_	13*T*_*h*_	2*T*_*ecm*_ + 7*T*_*h*_	4*T*_*ecm*_ + 34*T*_*h*_ + 1*T*_*FE*_
Meriam et al.^6^	4*T*_*ecm*_ + 8*T*_*h*_ + *T*_*E/D*_	2*T*_*ecm*_ + 5*T*_*h*_ + *T*_*E/D*_	2*T*_*ecm*_ + 2*T*_*h*_	8*T*_*ecm*_ + 15*T*_*h*_ + 2*T*_*E/D*_
Proposed scheme	5*T*_*ecm*_ + 22*T*_*h*_ + 1*T*_*FE*_	4*T*_*ecm*_ + 18*T*_*h*_	3*T*_*ecm*_ + 8*T*_*h*_	12*T*_*ecm*_ + 48*T*_*h*_ + 1*T*_*FE*_

From the computational time consumption in Table 4, it can be seen that the *T*_*FE*_ and *T*_*ecm*_ time consumption is high, and the *T*_*FE*_ of each scheme is similar, so the focus is on the point multiplication operation *T*_*ecm*_. This scheme uses the ECC-based key agreement scheme, and the point multiplication operation overhead is higher than that of other schemes, but it has higher security compared to other schemes that only use hash computation or symmetric encryption and decryption schemes. WSNs focus on the computational overhead of resource-constrained sensor nodes. The computational overhead of the sensor nodes is increased only once compared to schemes^6,39^, and^40^, which also have point multiplication operations. This scheme does not put too much pressure on sensor computation. Although the other schemes have less computational overhead, the present scheme is more effective in dealing with various security threats and is more suitable for high security systems.

### Communication overhead

**Figure 12:**
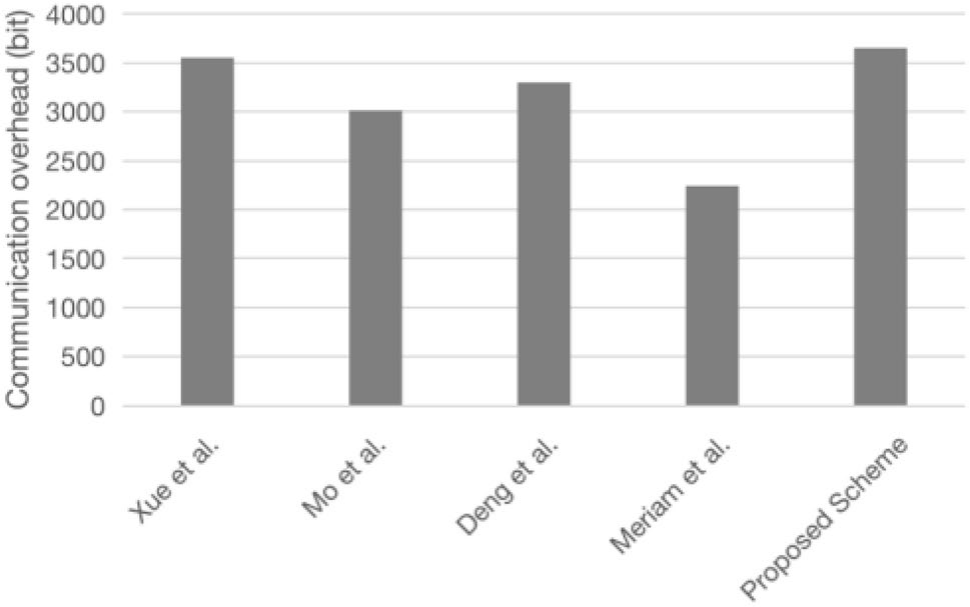
Total communication overhead comparison.

**Figure 13:**
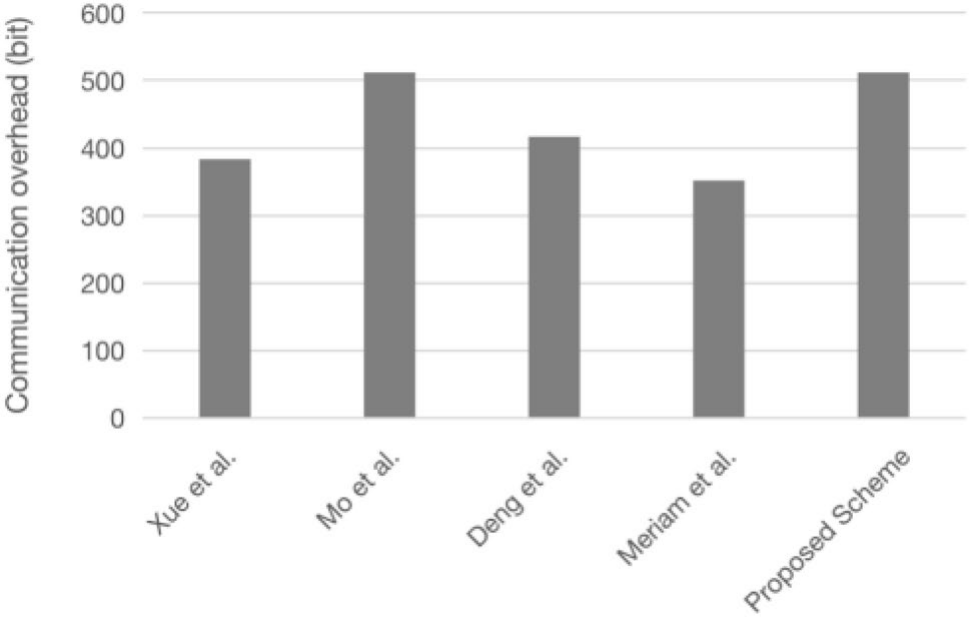
Comparison of node communication overhead.

The communication overhead is mainly for the data lengths of identity, hash value, fuzzy extractor public data, random numbers, timestamp, points of elliptic curve (public key), and symmetric encryption/decryption data. To facilitate the comparison, each data length in this scheme is set uniformly. The specific values are shown in Table 6, the comparison of communication overheads of each scheme is shown in Table 7, and the specific communication overhead quantization diagrams are shown in Figs. 12 and 13^41^.

**Table 6:** The notations, descriptions, and lengths required for communication data.

Notations	Descriptions	Length(bit)
*L*_*ID*_	Identity length	32
*L*_*h*_	Hash value length	160
*L*_*FE*_	Fuzzy extractor public data length	128
*L*_*r*_	Random number length	128
*L*_*T*_	Timestamp length	32
*L*_*ECC*_	Points of elliptic curve (public key) length	160
*L*_*E/D*_	Symmetric encryption/decryption data length	128

**Table 7:** Communication overhead comparison.

	*U*_*i*_	*GWN*	*S*_*j*_	Total
Xue et al.^16^	3*L*_*ID*_ + 1*L*_*FE*_ + 6*L*_*h*_ + 1*L*_*T*_	1*L*_*ID*_ + 1*L*_*FE*_ + 11*L*_*h*_ + 1*L*_*T*_	1*L*_*ID*_ + 2*L*_*h*_ + 1*L*_*T*_	5*L*_*ID*_ + 2*L*_*FE*_ + 19*L*_*h*_ + 3*L*_*T*_
Mo et al.^39^	1*L*_*ID*_ + 7*L*_*h*_ + 1*L*_*T*_	1*L*_*ECC*_ + 1*L*_*E/D*_ + 1*L*_*FE*_ + 5*L*_*h*_ + 3*L*_*T*_	1*L*_*ECC*_ + 2*L*_*h*_ + 1*L*_*T*_	1*L*_*ID*_ + 2*L*_*ECC*_ + 1*L*_*E/D*_ + 1*L*_*FE*_ + 14*L*_*h*_ + 5*L*_*T*_
Deng et al.^40^	1*L*_*ECC*_ + 5*L*_*h*_	2*L*_*ECC*_ + 10*L*_*h*_	1*L*_*ECC*_ + 2*L*_*FE*_	4*L*_*ECC*_ + 15*L*_*h*_ + 2*L*_*FE*_
Meriam et al.^6^	1*L*_*ECC*_ + 4*L*_*E/D*_ + 3*L*_*h*_ + 1*L*_*T*_	2*L*_*ECC*_ + 2*L*_*h*_ + 2*L*_*T*_	1*L*_*ECC*_ + 1*L*_*h*_ + 1*L*_*T*_	4*L*_*ECC*_ + 4*L*_*E/D*_ + 6*L*_*h*_ + 4*L*_*T*_
Proposed scheme	2*L*_*ECC*_ + 4*L*_*h*_ + 1*L*_*T*_	3*L*_*ECC*_ + 10*L*_*h*_ + 2*L*_*T*_	1*L*_*ECC*_ + 2*L*_*h*_ + 1*L*_*T*_	6*L*_*ECC*_ + 16*L*_*h*_ + 4*L*_*T*_

This scheme is based on ECC, and as the communication process needs to send each party’s public key several times, the communication overhead is slightly higher than with other schemes. For the communication overhead of resource-constrained sensor nodes, this scheme is the same as scheme^39^ and slightly higher than schemes^6,16^ and^40^, but still within the tolerance range of sensor nodes and suitable for WSNs.

## Conclusions

This paper examines multifactor authentication for WSNs. First, related schemes from recent years are introduced, and based on this, the scheme of Xue et al.^16^ is examined, with a focus on its advantages and security vulnerabilities. Then, a three-factor authentication and key agreement scheme based on ECC is proposed for WSNs. The security of the scheme is demonstrated by the BAN logical and informal analysis, and efficiency analysis shows that the scheme is used for resource-constrained WSNs. Overall, the proposed scheme effectively improves the security performance of WSNs based on efficiency and has good application value. Due to the use of ECC dot-multiplication operations, the computational energy consumption of the scheme is still higher compared to the scheme with only hash operations; therefore, in the next step of this research, the efficiency of the scheme needs to be further improved to guarantee security.

## Data Availability

The authors confirm that the data supporting the findings of this study are available within the article and its supplementary materials.
